# Hydrophobic Interactions between DNA Duplexes and Synthetic and Biological Membranes

**DOI:** 10.1021/jacs.0c13235

**Published:** 2021-05-20

**Authors:** Sioned F. Jones, Himanshu Joshi, Stephen J. Terry, Jonathan R. Burns, Aleksei Aksimentiev, Ulrike S. Eggert, Stefan Howorka

**Affiliations:** † Department of Chemistry, Institute of Structural and Molecular Biology, University College London, London WC1H 0AJ, United Kingdom; ‡ Randall Centre for Cell and Molecular Biophysics, School of Basic and Medical Biosciences, and Department of Chemistry, King’s College London, London SE1 1UL, United Kingdom; § Department of Physics and Beckman Institute for Advanced Science and Technology, University of Illinois at Urbana−Champaign, Urbana, Illinois 61801, United States; ∥ UCL Ear Institute, London WC1X 8EE, United Kingdom

## Abstract

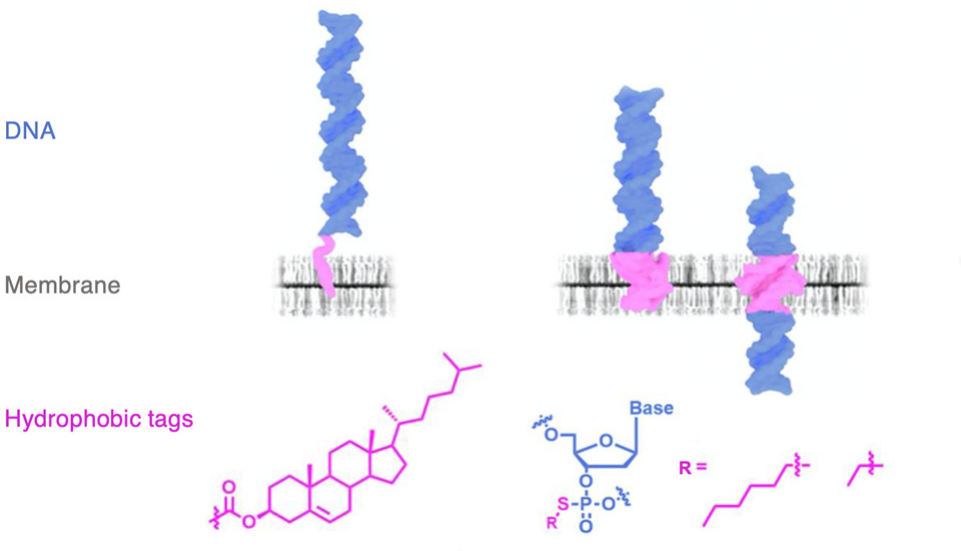

Equipping DNA with hydrophobic anchors enables targeted interaction with lipid bilayers for applications in biophysics, cell biology, and synthetic biology. Understanding DNA–membrane interactions is crucial for rationally designing functional DNA. Here we study the interactions of hydrophobically tagged DNA with synthetic and cell membranes using a combination of experiments and atomistic molecular dynamics (MD) simulations. The DNA duplexes are rendered hydrophobic by conjugation to a terminal cholesterol anchor or by chemical synthesis of a charge-neutralized alkyl-phosphorothioate (PPT) belt. Cholesterol-DNA tethers to lipid vesicles of different lipid compositions and charges, while PPT DNA binding strongly depends on alkyl length, belt position, and headgroup charge. Divalent cations in the buffer can also influence binding. Our MD simulations directly reveal the complex structure and energetics of PPT DNA within a lipid membrane, demonstrating that longer alkyl-PPT chains provide the most stable membrane anchoring but may disrupt DNA base paring in solution. When tested on cells, cholesterol-DNA is homogeneously distributed on the cell surface, while alkyl-PPT DNA accumulates in clustered structures on the plasma membrane. DNA tethered to the outside of the cell membrane is distinguished from DNA spanning the membrane by nuclease and sphingomyelinase digestion assays. The gained fundamental insight on DNA–bilayer interactions will guide the rational design of membrane-targeting nanostructures.

## Introduction

The unique properties of DNA duplexes enable precise engineering of nanostructures with different shapes, geometries, and sizes.^1−3^ Synthetic hydrophobic modifications expand the functional range of DNA nanostructures by facilitating specific interactions with lipid bilayers.^4−6^ The modifications include conjugating DNA to hydrophobic molecules such as cholesterol^7−13^ and porphyrin^14−16^ or a string of ethylated phosphorothioate (PPT) groups to generate a charge-neutralized DNA backbone.^17−19^ These hydrophobically tagged nanostructures advance the reorganization of membrane shape,^4,13,20−23^ the molecular transport across membranes,^9,10,24^ cell surface functionalization,^25,26^ and cytotoxicity.^18,27^ However, to realize the potential of the structures for diagnostics, therapeutics, and synthetic biology, a fundamental understanding of the interaction between hydrophobic DNA with lipid membranes is imperative.

Previous efforts to understand and improve nanostructure function at the membrane have been devoted to optimizing the interaction between cholesterol anchors and model membranes. Higher binding was obtained by increasing the number of anchors,^28,29^ improving their molecular accessibility by incorporating a flexible spacer chain, such as tetraethylene glycol (TEG),^30,31^ or by placing the anchors at exposed positions on the DNA structure.^28,29^ Advancements in atomistic molecular dynamics (MD) simulations have proven to be extremely useful in predicting the interactions of DNA nanostructures with lipid bilayers. MD simulations can explore structural dynamics and conformational flexibility to aid novel rational design strategies. This has been applied to study the strong tendency of cholesterol anchors to self-aggregate,^32^ the ethylation of PPT sites in nanopores and nanotubes,^33,34^ and the influence of lipid composition on the structure–function relationship of membrane-anchored DNA.^35^ In addition to the hydrophobicity of individual charge-neutralized nucleotides, their density within a DNA duplex can also influence the binding and insertion of DNA into a lipid bilayer membrane. Coarse-grained MD simulations found that the number of charge-neutralized DNA nucleotides can be as important as the length of the hydrophobic alkyl chains in stabilizing a patch of a lipid bilayer encircled by a DNA origami nanodisc.^36^ Another all-atom MD study of membrane-spanning nanopores^37^ found the density of the charges along the DNA backbone to affect the transmembrane transport of water and ions. While computational exploration of larger DNA–lipid constructs is currently possible using the all-atom MD approach,^38^ multiresolution simulation approaches^39^ are expected to bring out the best combination of computational efficiency and molecular realism.

Despite considerable progress in developing membrane-interacting DNA nanostructures, a systematic understanding of the interactions between hydrophobic DNA and lipid bilayers remains elusive. Fundamental questions are about the influence of anchor type on membrane binding affinity and distribution. What is the effect of increasing alkyl chain length of alkyl-PPT belt modifications and changing the belt position along the DNA? Furthermore, how does the interaction differ in synthetic vesicles of different compositions and live cell membranes? Does anchoring affect DNA and membrane structures, and how is binding governed by energetic factors? To understand the specific interaction between hydrophobic anchors and membranes, it is critical to use a reductionist approach whereby nanostructure size and geometry are kept constant to avoid additional contributing factors.

This report combines experiments and atomistic MD simulations to understand the complex interactions of hydrophobic DNA duplexes with lipid bilayers. We cover a broad range of hydrophobic anchors, differing in architecture, size, and position, to understand the design features that control membrane interaction. Anchoring efficiency is probed in various lipid environments, including giant unilamellar vesicles, simulated lipid bilayers, and live cell membranes. Our study will offer insight for the rational design of DNA nanostructures to advance their applications.

## Results and Discussion

### Design and Formation of Hydrophobic DNA

**Figure 1:**
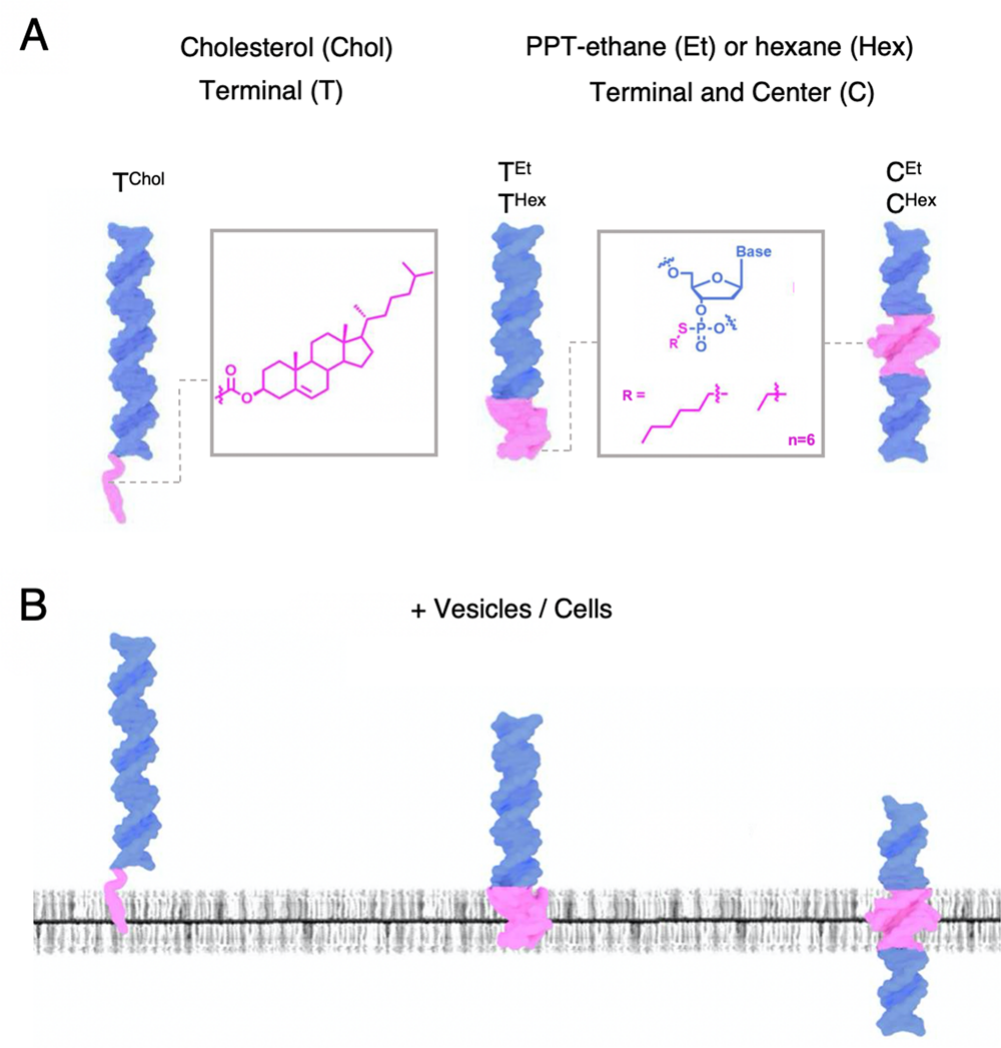
Hydrophobic dsDNA designed to interact with lipid bilayer membranes. (A) Lipid anchors for DNA strands. Cholesterol (chol) and alkyl-phosphorothioate (PPT). dsDNA construct T^Chol^ with a terminal cholesterol lipid anchor and constructs T^Et^ and T^Hex^ and C^Et^ and C^Hex^ carrying a terminal or central position alkyl-PPT belt. Each belt contains six pairs of PPT groups, fully alkylated with ethyl or hexyl groups. (B) Hydrophobic modifications (magenta) allow DNA T^Chol^ to tether to the membrane while DNA duplexes T^Et^ and T^Hex^, and C^Et^ and C^Hex^, can penetrate and span the bilayer, respectively.

A 30 base pair DNA duplex served as the probe nanostructure. Several hydrophobic modifications were used. In the construct T^Chol^, the duplex is rendered hydrophobic by a TEG-linked cholesterol anchor at the 3′ terminus of the two-component single-stranded DNA (ssDNA) (Figure 1A). T^Chol^ is expected to tether to the lipid bilayer (Figure 1B). The other duplex constructs feature a charge-neutralized DNA backbone segment of alkyl-PPT (Figure 1A), used previously in DNA nanopores.^17,18^ Each duplex contains six pairs of PPT groups charge-neutralized with ethyl or hexyl modifications. The duplexes with hydrophobic belts at the terminal or central position, T^Et^ and T^Hex^, and C^Et^ and C^Hex^ (Figure 1A), are expected to penetrate and span the lipid bilayer (Figure 1B). A fluorescent Cy3 tag at the 3′ terminus of one ssDNA in the duplex allows for detection by fluorescence microscopy (Table S1 for DNA sequences).

**Figure 2:**
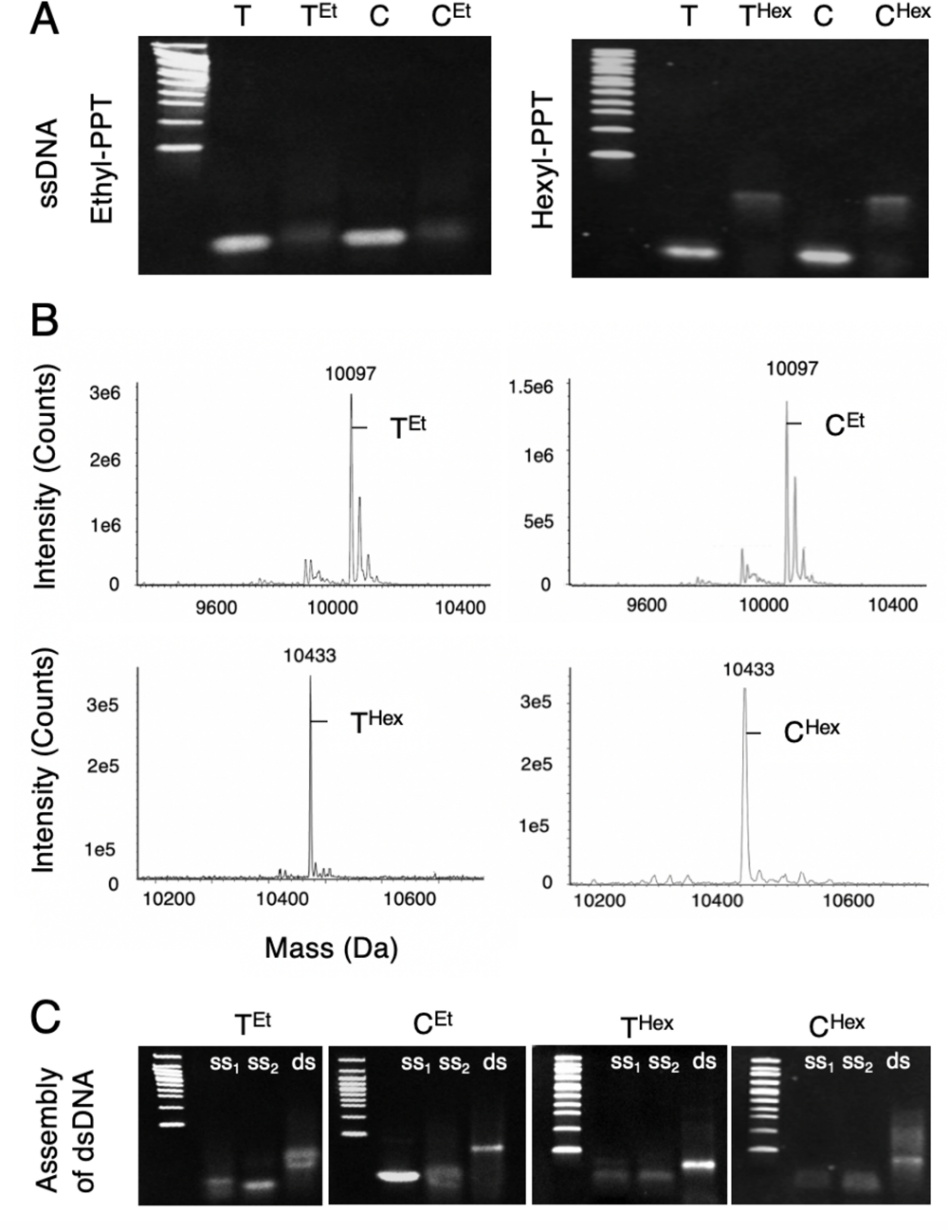
Characterization of ssDNA and dsDNA carrying alkyl-PPT modifications. (A) Sodium dodecyl sulfate (SDS) PAGE analysis of terminal (T) and central (C) PPT-containing ssDNA with and without ethyl (Et) or hexyl (Hex) modifications. A 100 bp ladder is shown on the left of each gel. Bands were visualized by ethidium bromide. (B) UPLC-MS analysis of ethyl and hexyl-PPT ssDNA at the terminal and central position. The peaks at 10 097 and 10 433 Da indicate complete modification with ethyl and hexyl of all PPT sites in a Cy3-labeled ssDNA. (C) SDS PAGE analysis of the assembly of all alkyl-PPT dsDNA formed by hybridization of two alkyl-PPT ssDNAs (ss1 and ss2).

The DNA strands for construct T^Chol^ were obtained from a commercial source, while ssDNAs for T^Et^, T^Hex^, C^Et^, and C^Hex^ were prepared by subjecting PPT-containing oligonucleotides to nucleophilic substitution with iodoalkane to yield alkyl-PPT moieties. The addition of ethyl and hexyl groups on ssDNA was established in polyacrylamide gel electrophoresis (PAGE) via a gel shift (Figure 2A). Ultra-high-performance liquid chromatography–mass spectrometry (UPLC-MS) also confirmed alkylation of all PPT positions on the ssDNA (Figure 2B). The hydrophobic DNA duplexes were assembled by hybridizing an equimolar amount of ssDNA and complementary ssDNA, as monitored by PAGE analysis (Figure 2C). Dynamic light scattering yielded a duplex size of ∼5 nm, with the exception of T^Chol^ (∼7 nm) due to the TEG linker and anchoring group. Polydispersity values of 25–35% suggested mainly monodisperse samples (Table S2).

### DNA–Membrane Binding to Synthetic Membranes Is Influenced by the Hydrophobic Anchor

**Figure 3:**
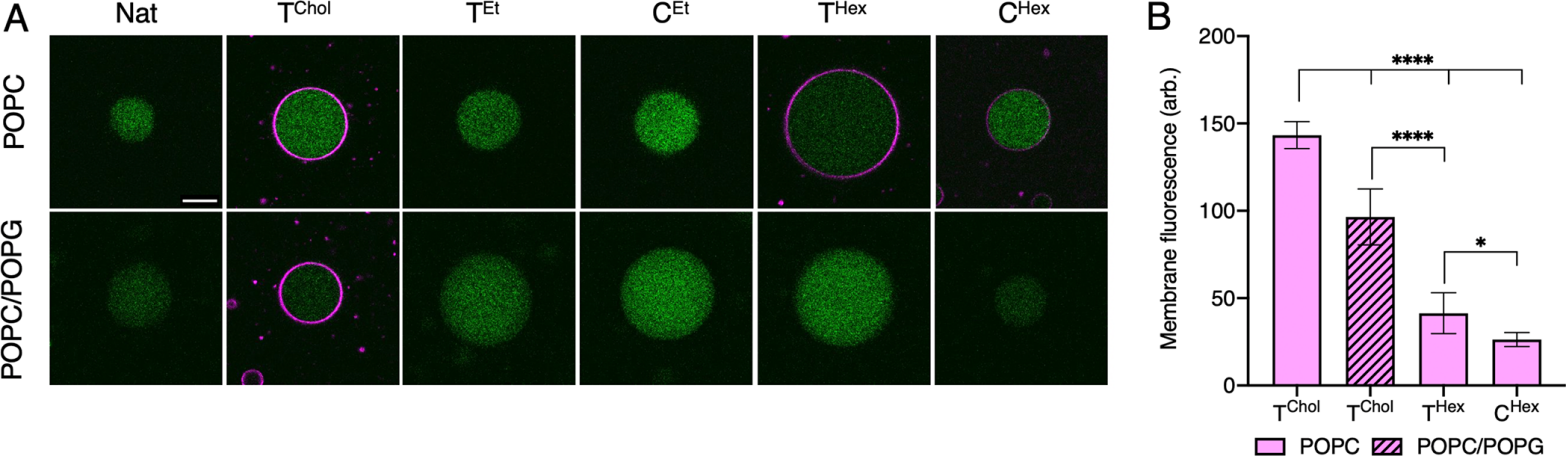
DNA–membrane interaction in synthetic membranes is influenced by architecture, size, and position of the hydrophobic modification and lipid bilayer composition. (A) Fluorescence confocal microscopy analysis of dsDNA (1 μM) in PBS buffer with GFP-encapsulated GUVs following 5 min of incubation. GUVs are composed of POPC or 1:1 POPC/POPG lipids. The panels present the overlay of GFP (green) and Cy3 (purple) channels. Representative images from three independent experiments are shown. The intensity of the encapsulated GFP can vary and has been adjusted for display purposes. The Cy3 images were collected and processed identically. Scale bar, 10 μm. (B) Plot summarizing the relative Cy3-DNA membrane fluorescence intensities from the panels in A. The data are presented as mean ± SD collected from three independent experiments, *n* = 10 GUVs per condition. One-way ANOVA using Tukey’s multiple comparisons test (*P = 0.0178, ****P < 0.0001).

We examined the interactions of different hydrophobic anchors with giant unilamellar vesicle (GUV) membranes using confocal fluorescence microscopy. To aid visualization, green fluorescent protein (GFP) was encapsulated in GUVs. Vesicles with the net neutrally charged lipid 1-palmitoyl-2-oleoyl-glycero-3-phosphocholine (POPC) were examined first. Incubation of GUVs with the hydrophobic duplexes in PBS or Opti-MEM cell culture medium followed by imaging yielded Cy3 fluorescent signal for constructs T^Chol^, T^Hex^, and C^Hex^ (Figures 3A, S1), indicative of binding. In quantitative analysis, T^Chol^ was strongest, followed by T^Hex^, and C^Hex^ (Figure 3B). Membrane binding was also influenced by the position of the hexyl belt, with the terminal belt better than a central one. No binding was observed for the native Cy3-labeled duplex (Nat) without hydrophobic modifications. Binding of tagged DNA was influenced by the lipid headgroup as tested with GUVs composed of 1:1 POPC and negatively charged 1-palmitoyl-2-oleoyl-*sn*-glycero-3-phospho-(1′-rac-glycerol) (POPG) lipids. Membrane binding was significantly reduced for T^Chol^, while T^Hex^ and C^Hex^ did not bind, likely due to electrostatic repulsion between the phosphate backbone and the POPG lipids.^35^ The weak binding of T^Chol^ may be attributed to the TEG linker, which could help reduce the electrostatic repulsion. Nevertheless, divalent cations can overcome the repulsion. Adding MgCl_2_ (10 mM) to PBS led to weak binding of T^Hex^ and C^Hex^ to POPC/POPG GUVs and a higher affinity for T^Chol^ (Figure S2). The use of divalent ions to strengthen the DNA–membrane interaction is known^40,41^ and has been used to adsorb large DNA origami units to zwitterionic lipid bilayers.^42^ T^Et^ and C^Et^ did not bind to any GUVs, even when tested under increased DNA concentration, higher ionic strength, or longer incubation time (data not shown). No binding occurred for PPT strands lacking the alkyl modification even when the pH was dropped below the p*K*_a_ of the thiol group of PPT (data not shown).^43^

We investigated the influence of membrane order and phase on DNA interactions with GUVs in PBS. Membrane order was tested by changing cholesterol content from zero to a ratio of 1:1 and 1:2 POPC/Chol. Confocal microscopy images (Figure S3) show that 1:1 POPC/Chol increased affinity of T^Chol^ significantly compared to POPC, while 1:2 POPC/Chol reduced it. Binding of T^Hex^ and C^Hex^ was also decreased at higher cholesterol content. To determine the influence of membrane phase, we tested 1:1 POPC/DPPC GUVs (Figure S4). DPPC (1,2-dipalmitoyl-*sn*-glycero-3-phosphocholine) has a phase transition temperature of 41 °C, allowing the formation of gel phase membranes. The binding affinity of T^Hex^ duplexes was substantially reduced in POPC/DPPC GUVs. This could suggest more favorable interaction with liquid phase bilayers in PBS buffer. Our findings highlight that the type and position of hydrophobic anchor, ionic buffer conditions, and membrane composition can influence DNA interactions with synthetic lipid bilayers.

### MD Simulations Reveal the Structure and Energetics of Membrane-Embedded Alkyl-PPT Modifications

To elucidate the varied molecular interactions between alkyl-modified dsDNA and the lipid bilayer membrane, we constructed six all-atom models of a 30 base pair dsDNA modified with either ethyl, butyl, or hexyl PPT groups. Each system contained six pairs of alkyl-PPT groups to form a hydrophobic belt that was introduced either at the central or at the terminal position of the dsDNA molecule, in line with the experimental data set.

**Figure 4:**
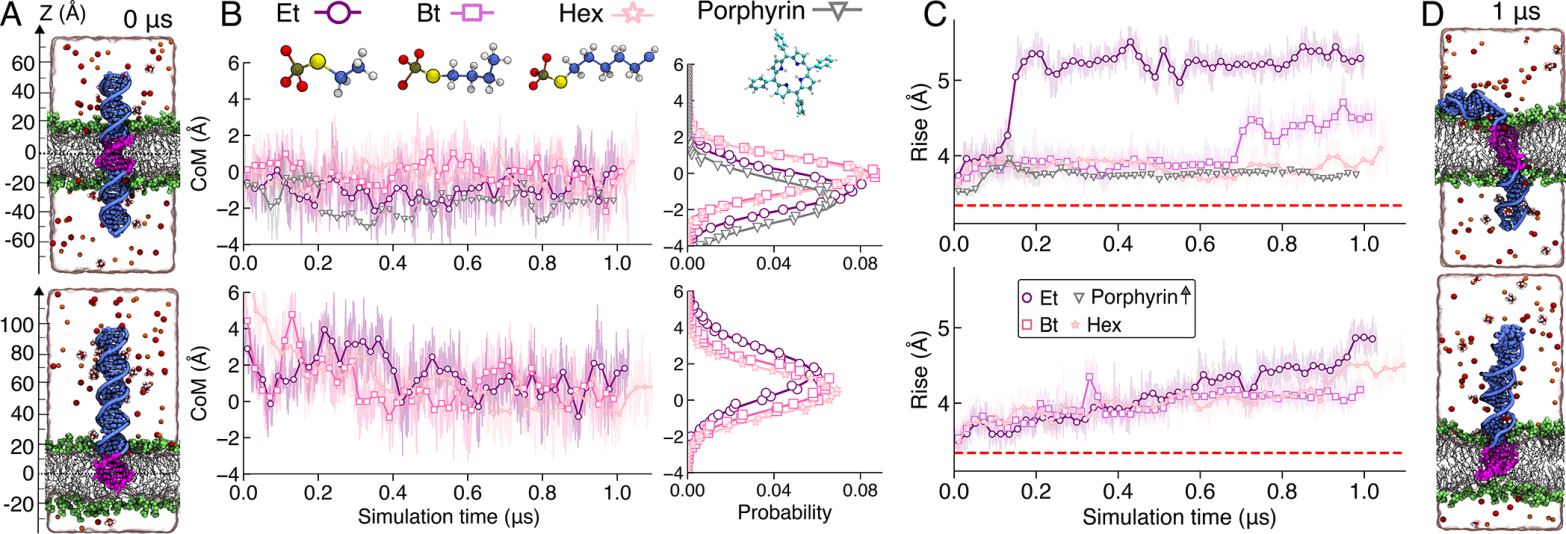
Molecular dynamics simulations of alkyl-modified DNA embedded in a lipid membrane. (A) Cut-away views of the centrally (top) and terminally (bottom) anchored DNA systems at the beginning of the MD runs. The modified nucleotides of the DNA are shown in magenta; the unmodified, in blue. The non-hydrogen atoms of the POPC lipid headgroups are shown as green spheres, whereas the lipid tails are shown as white lines. The volume occupied by 150 mM NaCl electrolyte is represented by a semitransparent surface; select sodium (red), chloride (orange), and magnesium hexahydrate (red and white) ions are explicitly shown. (B) *Z* coordinate of the modified DNA center of mass as a function of simulation time (left). Each symbol represents a 20 ns block average; the instantaneous trace is shown in the background. The distribution of the *Z* coordinate (right). The data analysis excluded the first 200 ns of each trajectory. Each line shows the Gaussian fit to the data (0.2 Å bin size). (C) Average base-pair rise of the modified DNA as a function of simulation time for centrally (top) and terminally anchored (bottom) systems. The dotted red line indicates the standard rise of B-DNA (3.32 Å). The gray line shows the average rise for porphyrin-anchored DNA taken from a previously published study.^44^ (D) Final configurations of the ethyl-modified DNA systems; Figure S5 shows final configurations for all simulated systems.

The molecules were embedded into a POPC lipid bilayer membrane and submerged in a 150 mM solution of NaCl. Table S3 lists all simulations performed in this study. Figure 4A illustrates the initial configuration of the simulated systems. After a brief restrained equilibration, the systems were simulated without any restraints for 1 μs using the MD method. Supplementary Movies 1 and 2 illustrate these simulation trajectories, whereas Figure S5 shows the final configuration of each system.

To characterize the placement of the modified DNA within a lipid membrane, we plotted the center of mass (CoM) coordinate of the modified DNA fragment as a function of simulation time for both centrally and terminally anchored constructs (Figure 4B). For comparison, we also characterized the placement of a porphyrin-modified dsDNA using previously reported MD trajectories.^44^ For all types of constructs, the modified nucleotides were found to locate near the center of the lipid membrane (at *Z* = 0 Å), although some statistically significant deviations were apparent. The standard deviation of the CoM coordinate from its average was 1.05, 0.93, and 0.98 Å for centrally modified ethyl, butyl, and hexyl systems, respectively, and broader, 1.36 (ethyl), 1.23 (butyl), and 1.16 (hexyl) Å, for terminally anchored systems. As the width of the distribution qualitatively reports on the strength of the anchoring interactions, we conclude that centrally modified constructs provide a more stable anchoring than the terminally modified ones, provided that they both can embed into a lipid bilayer membrane.

The type and the placement of DNA nucleotide modifications were found to profoundly affect the local structure of the DNA duplex (Figure 4C). The average rise (distance between consecutive base pairs) in the porphyrin-modified DNA was closest to that of a canonical B-form DNA likely because of the abundant hydration of the modified duplex inserted in a lipid bilayer.^44^ Centrally anchored alkyl-modified DNA were observed to stretch within the bilayer, with the stretching being the most prominent in the case of ethyl-modified DNA (Figure 4D) and the least for the hexyl-modified one. Pronounced local stretching was previously observed in MD simulations of cholesterol-modified DNA constructs spanning through a lipid membrane.^45^ A smaller degree of stretching was observed in the terminally modified DNA systems, although our simulations, likely, did not reach equilibrium configurations. Stretching of the modified DNA fragments was accompanied by partial deterioration of the base-pairing interactions (Figure S6). As expected, unmodified DNA was observed to completely escape from the lipid membrane within approximately 200 ns (Figure S7 and Movie 3). Similarly, a DNA construct terminally attached to a membrane via a cholesterol anchor remained in the solution, showing negligible structural distortions (Figure S7).^35^ Interestingly, the local stretching of ethyl-modified DNA within a lipid membrane was considerably smaller when the DNA molecules were arranged into a six-helix bundle (Figure S8), likely because of water filling the nanopore at the center of the bundle.^37^ Experimental verification of alkyl-PPT stretching could not be conducted due to the complexity of detecting nanometer and sub-nanometer scale structural changes occurring within a six-bp PPT region embedded in a dynamic lipid bilayer.

**Figure 5:**
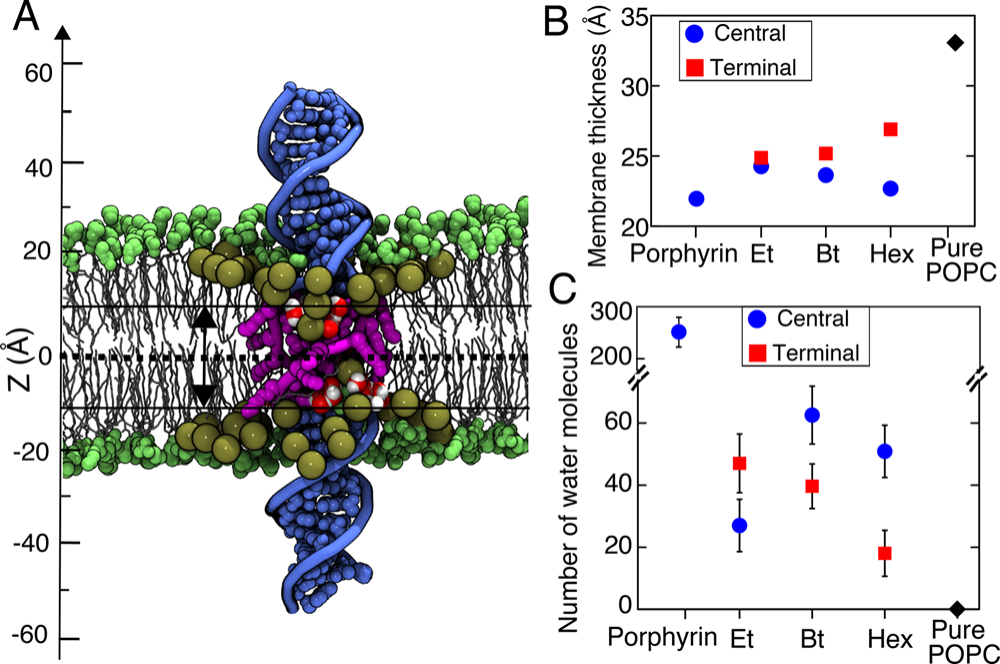
Local structure of the lipid membrane surrounding modified DNA. (A) Cut-away view of a hexyl-modified DNA system after 800 ns of MD simulation. The C2 atoms of the lipid molecules located within 15 Å of the DNA (used to compute the local membrane thickness) are shown as large tan spheres, whereas all other headgroup atoms are illustrated using smaller green spheres. Water molecules located within 10 Å from the midplane of the lipid bilayer are shown using red (oxygen) and white (hydrogen) spheres. The modified nucleotides of the DNA are shown in magenta; the unmodified, in blue. (B) Local thickness of the lipid membrane surrounding the DNA constructs measured as the distance between the peaks of the C2 atom density. (C) Number of water molecules present within 10 Å of the membrane’s midplane averaged over the respective MD trajectories (excluding the first 200 ns). Error bars show standard deviations of 2 ns sampled data.

We characterized the effect of DNA anchoring on the local structure of the lipid bilayer by computing the local thickness of the lipid membrane surrounding the DNA and the number of water molecules located within the hydrophobic plane of the bilayer (Figure 5A). For all types of DNA modifications, the lipid membrane was found to be considerably thinner in the vicinity of the DNA (Figure 5B). A porphyrin-anchored DNA duplex was previously reported to form a water-filled toroidal pore surrounding the DNA helix.^44^ While we did not observe a continuous water-filled nanopore surrounding the alkyl-modified DNA systems, a substantial number of water molecules were found bound to the DNA duplexes (Figure 5C).

**Figure 6:**
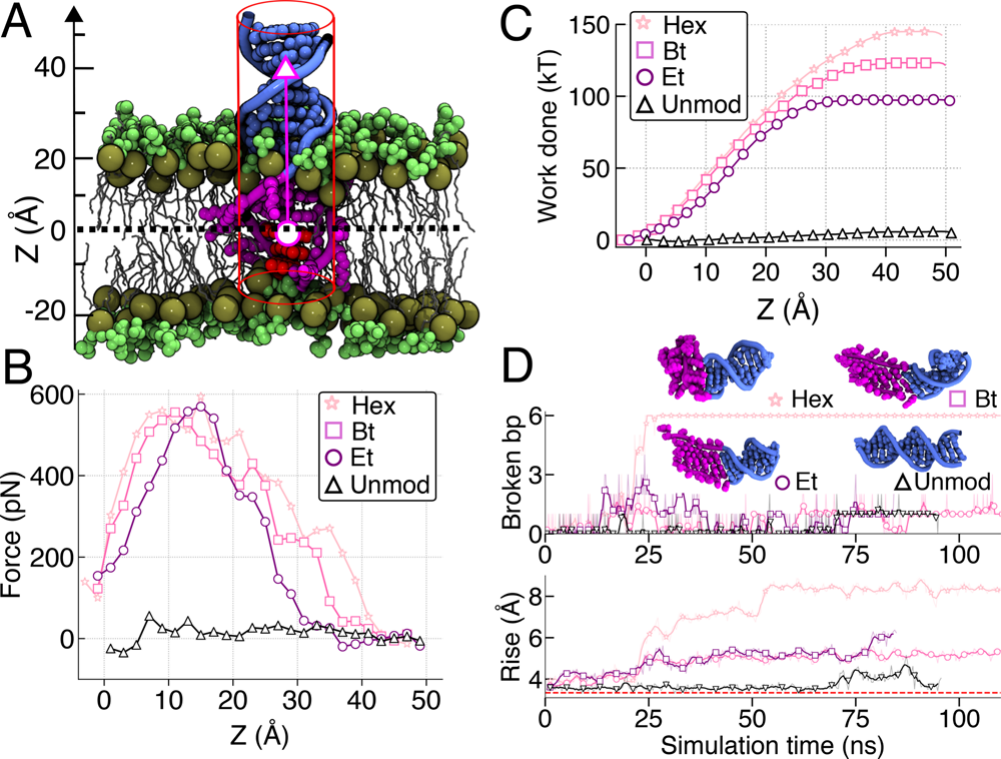
Pulling out anchored DNA using external force. (A) Schematics of the simulations. The SMD template particle (magenta circle) is moved away from the lipid membrane with a constant velocity of 0.5 Å/ns. The CoM of the terminal three base pairs (red) is linked to the template particle via a harmonic potential. The phosphorus atoms of the DNA are restrained to remain at the surface of a cylinder (red lines). The C2 atoms of the lipid molecules (tan spheres) are harmonically restrained to their initial *z* coordinates. (B) The average force exerted on the DNA as a function of the CoM *z* coordinate of the three terminal base pairs. The force was averaged in 2 Å bins. (C) Work done by the SMD force versus the CoM *z* coordinate of the three terminal base pairs. (D) Number of broken base pairs (top) and the average rise (bottom) of the six terminal base pairs of the 30 base pair constructs during their equilibration in electrolyte solution. The snapshots illustrate the conformations of the DNA constructs at the end of the simulations.

To directly probe the effect of DNA modification on the strength of lipid anchoring, we used the steered molecular dynamics (SMD) method^46,47^ to pull the DNA out of the bilayer (Figure 6a and Supplementary Movie 4). As the modified DNA is pulled away from the membrane, the pulling force increases until the modified DNA leaves the membrane (Figure 6B). Whereas the maximum SMD force has a similar value for all three modifications, the force rises more sharply for more hydrophobic modifications, which results in a substantial difference in the work performed by the SMD spring to pull the modified DNA completely out of the lipid membrane (Figure 6C). In contrast, the unmodified DNA requires close to zero external force to be pulled out of the membrane and hence close to zero work. While the absolute values of the work done during the pulling process are expected to considerably exceed the equilibrium free-energy differences between membrane-anchored and electrolyte solution states,^48^ the qualitative ranking suggests more favorable binding of more hydrophobic DNA constructs.

Close examination of the SMD trajectories (Supplementary Movie 4) reveals that the structure of modified DNA exhibits significant deviations from that of an ideal B-form helix after being completely out from the membrane. To determine if such structural deformations occur in the absence of the external forces, we ran free equilibration simulation of the terminally modified DNA constructs in the electrolyte solution. Analysis of the simulation trajectories (Figure 6D) indicates significant instability of the modified DNA fragments that increases with the hydrophobicity of the modifications. The structure of hexyl-modified DNA becomes particularly distorted, with all six base pairs coming apart. This highlights that increased hydrophobicity of alkyl chain helps retain the structure in the membrane and provides the most stable anchoring. However, this can profoundly affect the structure of the DNA and may disrupt DNA base paring in solution.

### Differential Interaction of DNA Duplexes with Biological Membranes of Live Cells

**Figure 7:**
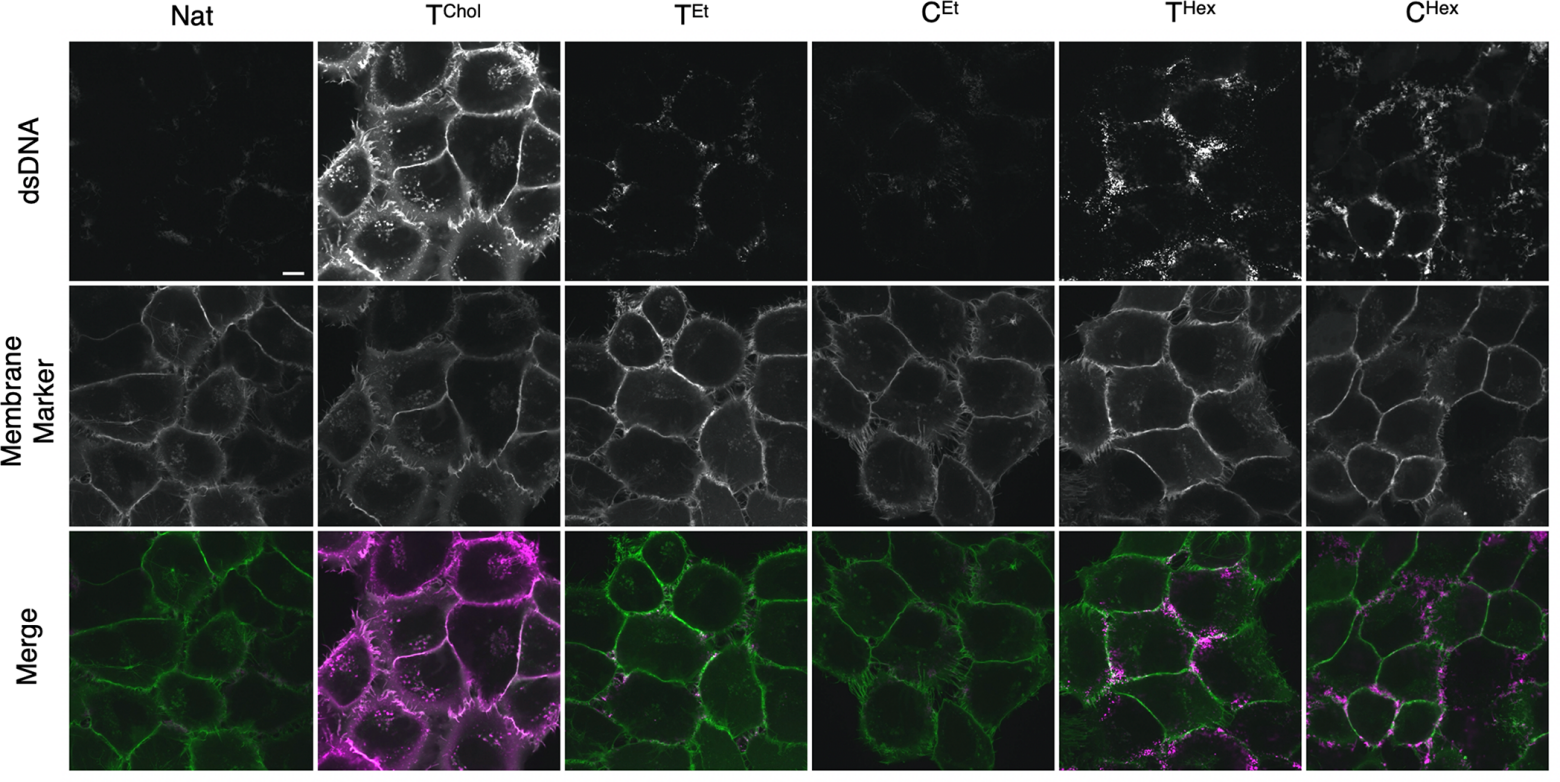
Hydrophobic DNA duplexes bind to the plasma membrane of live HeLa cells and demonstrate anchor-specific interactions. Confocal fluorescence microscopy images of live MyrPalm-EGFP HeLa cells treated with hydrophobic dsDNA (0.2 μM) in Opti-MEM for 5 min. The fluorescently labeled DNA (top panels, magenta in merge), membrane marker (MyrPalm-EGFP, middle panels, green in merge), and a merge of both channels (bottom panels) are shown. Representative images from three independent experiments are shown; *n* = 300 cells analyzed per condition. All images were collected and processed under identical conditions. Scale bar, 10 μm.

To elucidate DNA–membrane interactions in cellular bilayers, we tested the DNA duplexes on live human cultured cells frequently used in DNA nanotechnology.^11,49,50^ A HeLa cell line was genetically engineered to stably express a GFP membrane marker (MyrPalm-EGFP) to allow visualization by fluorescence microscopy. Prior to incubation with DNA, the cells were washed in serum-free Opti-MEM medium to remove serum proteins that can nonspecifically adhere to DNA nanostructures and interfere with cell binding.^11,51,52^ Cells were incubated with DNA duplexes in Opti-MEM for 5 min, washed, incubated with fresh Opti-MEM, and imaged by confocal fluorescence microscopy (Figure 7). Control native DNA (Nat) gave rise to a weak fluorescent signal at the plasma membrane, likely due to the hydrophobic Cy3 fluorophore interacting with the plasma membrane. This is different from lack of binding to GUVs, likely as cell membranes have a more complex lipid and protein composition.

Lipid-tagged duplex T^Chol^ displayed a bright fluorescent signal that strongly colocalized with the plasma membrane marker (Figure 7). Quantification of the fluorescence signal by the Manders coefficient analysis (Figure S9) indicated a homogeneous distribution of T^Chol^ over the entire plasma membrane. Duplexes T^Hex^ and C^Hex^ as well as T^Et^ and C^Et^ also associated with live HeLa cells, yet were weaker (Figure 7) and less homogeneous than T^Chol^ (Figure S9). Similar DNA–membrane interaction patterns were also observed in live human bone osteosarcoma cells (U2OS) (Figure S10). We note that the cell-binding data are different from the GUV results, where interaction was found only for T^Hex^ and lesser for C^Hex^.

Fluorescent clusters were seen on T^Hex^- and C^Hex^-incubated cells (Figure 7). Higher-order structures could arise from hexyl belts’ interaction with neighboring duplexes on the biological membranes. T^Hex^ clusters were larger than those of C^Hex^, possibly due to the more amphiphilic nature of hexyl tags and the higher likelihood to form micelle-like structures. No aggregation was found in aqueous environments, suggesting the preferential binding of DNA to specific regions within the plasma membrane.^53^ The distribution patterns of hydrophobic DNA may also be influenced by different membrane regions, as has been observed in some cell types.^26,54^ Our work on synthetic bilayers shows that alkyl-PPT DNA duplexes are less likely to insert into membranes composed of ordered and gel phase lipids (Figures S3, S4), suggesting that alkyl-PPT DNA–membrane recognition is influenced by membrane composition. Future work is required to fully understand the interaction of hydrophobic DNA with the complex structures that make up live cell membranes.

After incubation and membrane binding, DNA duplexes remained on the plasma membrane of live cells up to 30 min for ethyl-PPT and 60 min for hexyl-PPT and T^Chol^ at 37 °C (Figure S11). PPT modification also minimized cellular internalization to after 60 min, while T^Chol^ entered cells immediately after incubation.

We distinguished between DNA tethered to the cell membrane and DNA spanning the membrane by measuring the accessibility of a nuclease.^11,55^ In the absence of membranes, DNase I degrades all DNA duplexes as shown by ethidium bromide staining and gel electrophoresis (Figure S12). Some remaining DNA bands stem from the lower DNase I activity for ssDNA than dsDNA. The similar intensities of remaining gel bands suggest that DNA duplexes with different hydrophobic tags are digested by DNase to the same extent. Similar results were found when detecting the Cy3 signals in gels via fluorescence scanning without ethidium bromide staining (Figure S12). The Cy3 signal colocalized to fragmented DNA and was similar for all constructs. Digestion by DNase I also led to a fluorescence emission reduction (Figure S13), as cleaved off Cy3 can bind to nucleobases or self-quench.^55^

**Figure 8:**
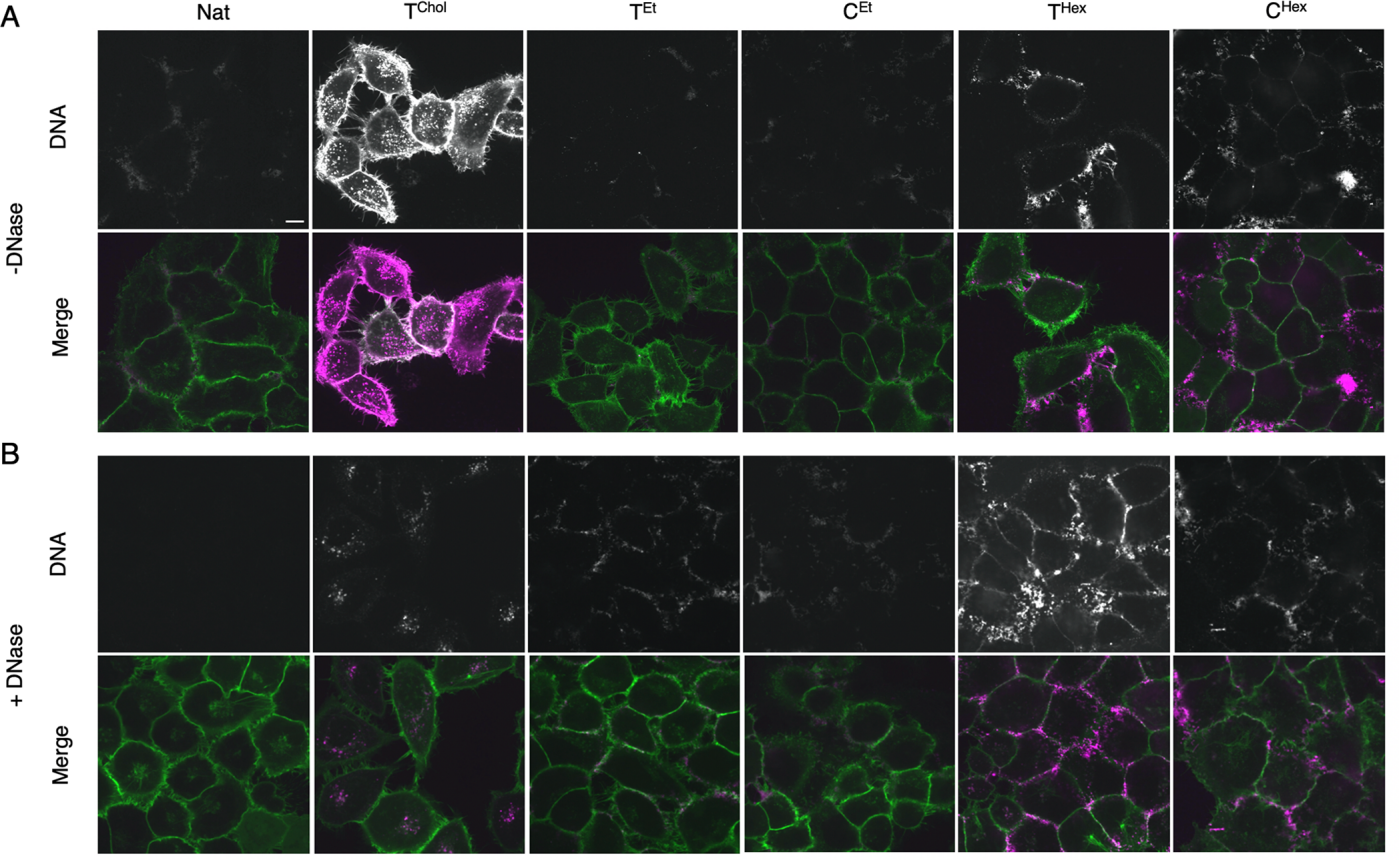
Nuclease digestion assay can distinguish between membrane-tethering and membrane-spanning orientations of dsDNA. Confocal fluorescence microscopy images of MyrPalm-EGFP HeLa cells bound to DNA (A) and following incubation with DNase (I) (20 U/mL) for 10 min (B). Images of A and B were taken at the same time point (10 min). The top panels in both A and B show the DNA, and the bottom panels show the overlay of DNA and the membrane marker (DNA is magenta and the membrane marker is green). Representative images from three independent experiments are shown. All images were collected and processed under identical conditions. Scale bar, 10 μm.

When DNA-bound cells were treated with DNase I, the membrane fluorescence associated with T^Chol^ and Nat was lost, leaving only the internalized fluorescence visible (Figures 8 and S14). Conversely, the membrane fluorescence of alkyl-PPT duplexes was unaffected by DNase I treatment. Similar results were obtained with sphingomyelinase (SMase), which digests sphingomyelin on the outer leaflet of the membrane and results in disruption of the leaflet. Treatment with SMase for 30 min resulted in the loss of Nat and T^Chol^ fluorescence but not alkyl-PPT DNA (Figures S15, S16), strengthening our hypothesis that alkyl-PPT DNA spans both leaflets of the bilayer.

## Conclusion

We have conducted a comparative study to investigate the interaction of hydrophobic duplexes with different hydrophobic modifications in lipid vesicles, simulated bilayers, and live cell membranes. In examining their anchoring efficiency in vesicles, we discovered that cholesterol-tagged DNA interacted with membranes more strongly than alkyl PPT duplexes, likely due to hydrophobicity of the cholesterol lipid group and its accessibility to the membrane due to its terminal anchoring. For DNA containing alkyl-PPT, the position of the PPT groups and length of the alkyl chains were relevant in determining membrane binding, whereby a hexyl-PPT belt was required to drive membrane insertion. Our data indicate that a negative membrane charge can repel DNA duplexes and decrease binding affinity in PBS buffer. However, this can be partly overcome by the addition of divalent cations such as MgCl_2_. The anchors must exhibit a strong hydrophobic effect to overcome repulsion. Binding is also influenced by order and phase in the lipid bilayer when tested in PBS buffer.

Our MD simulations painted a complex picture of how the alkyl-PPT design features (alkyl chain length and PPT belt position) influence the structure and energetics of DNA anchoring within a membrane. On one hand, increased hydrophobicity of modified nucleotides allows for retaining the structure of the DNA double helix in the lipid bilayer environment and provides the most stable membrane anchoring. Reducing the hydrophobicity of the modifications results in local stretching of the DNA, which occurs to minimize the free-energy penalty associated with the insertion of a polar molecule within a hydrophobic environment of a lipid membrane. Interestingly, even partial hydration of such modifications reduces the amount of stretching, as seen in the case of DNA nanopore bundles. On the other hand, the presence of hydrophobic modifications profoundly affects the structure of the modified DNA in an aqueous solution environment to the point that base-pairing interactions no longer can ensure complementary binding of the two DNA strands into a DNA duplex. The optimal choice of the modification will ultimately depend on the intended function of the design and will require a compromise between the efficiency of lipid membrane insertion and the ability to fold into the prescribed shape in aqueous solution.

When extending our study to live HeLa cells, we demonstrated differences between DNA interactions in synthetic and biological membranes. All duplexes interacted with the plasma membrane of live cells, including the native DNA with a Cy3 tag. This highlights the complexity of cell membranes, compared to simplified GUVs and simulated bilayers. Conducting experiments in both synthetic and biological lipid environments is therefore crucial in understanding the interaction of DNA nanostructures with membranes. While the cholesterol DNA adhered homogeneously to cell membranes, alkyl-PPT DNA gave rise to heterogeneous distributions at the membrane and assembled to higher-order structures. The tethering nature of T^Chol^ to the outer membrane leaflet and the membrane-spanning orientations of alkyl-PPT duplexes were suggested using nuclease and sphingomyelinase digestion assays. The design principles presented in this study can be applied to rationally design DNA nanostructures and achieve selective interaction with lipid bilayers in synthetic and biological systems to advance applications.

## Supporting Information Available

Detailed experimental methods, table of DNA sequences, additional confocal microscopy images with analysis, and MD simulations (PDF)
Additional supplementary MD movie (AVI)
Additional supplementary MD movie (AVI)
Additional supplementary MD movie (AVI)
Additional supplementary MD movie (AVI)

## Supplementary Material

ja0c13235_si_001.pdf

ja0c13235_si_002.avi

ja0c13235_si_003.avi

ja0c13235_si_004.avi

ja0c13235_si_005.avi
