# Supplementary material for: Hydrophobic Interactions between DNA Duplexes and Synthetic and Biological Membranes
Source: J Am Chem Soc. 2021 May 20;143(22):8305–13. doi: 10.1021/jacs.0c13235 (PMC8193631; doi:10.1021/jacs.0c13235)
Supplement: Supplementary file 1 — ja0c13235_si_001.pdf [file ja0c13235_si_001.pdf]

SUPPORTING INFORMATION

Hydrophobic interactions between DNA duplexes and synthetic and biological membranes

Sioned F. Jones,[a,b] Himanshu Joshi,[c] Stephen J. Terry,[b] Jonathan R. Burns,[a] Aleksei Aksimentiev,[c] Ulrike S. Eggert[b] and Stefan Howorka[a*]

a Department of Chemistry, Institute of Structural and Molecular Biology, University College London, London WC1H 0AJ, United Kingdom

b Randall Centre for Cell and Molecular Biophysics, School of Basic and Medical Biosciences, and Department of Chemistry, King's College London, London, SE1 1UL, United Kingdom

c Department of Physics, and Beckman Institute for Advanced Science and Technology, University of Illinois at Urbana–Champaign, Urbana, Illinois 61801, United States

d UCL Ear Institute, London, UK WC1X 8EE, United Kingdom

Table of contents:

1. Experimental Section 2

1.1. Materials 2

1.2. Synthesis of Alkyl-Phosphorothioate DNA Backbone Modification 2

1.3. Assembly of Hydrophobic DNA Duplexes 2

1.4. Size Determination Using Dynamic Light Scattering (DLS) 2

1.5. Analysis of DNA-Membrane Interaction in Vesicles using Fluorescence Microscopy 2

1.6. Atomistic Molecular Dynamics (MD) Simulations 3

1.7. Plasmid DNA Construction and Production of Stable MyrPalm-EGFP HeLa Cell Line 4

1.8. Cell Culture 4

1.9. Analysis of DNA-Membrane Interaction in Live Cells using Fluorescence Microscopy 4

1.10. Nuclease Digestion of Membrane-Bound DNA Duplexes 5

1.11. Sphingomyelinase treatment of DNA-bound cells. 5

2. Supplementary Tables and Figures 6

3. Captions to Supplementary Movies 18

4. References 21

1. Experimental Section

1.1. Materials

All DNA oligonucleotides were purchased from Integrated DNA Technologies (Iowa, USA), with HPLC purification. 1-palmitoyl-2-oleoyl-glycero-3-phosphocholine (POPC), 1-palmitoyl-2-oleoyl-sn-glycero-3-phospho-(1'-rac-glycerol) (sodium salt) (POPG), 1,2-dipalmitoyl-sn-glycero-3-phosphocholine (DPPC) and cholesterol were purchased from Avanti Polar Lipids (AL, USA). All other reagents and solvents were purchased from Merck (Darmstadt, Germany) or Fischer Scientific (UK) unless otherwise stated.

1.2. Synthesis of Alkyl-Phosphorothioate DNA Backbone Modification

Alkyl groups of varying carbon chain length were introduced to a phosphorothioate (PPT)-backbone ssDNA using a modified version of a protocol by Burns *et al.*[1-2] PPT-containing ssDNA (5 nmol) was dissolved in 90 % dimethylformamide (DMF) and 10% tris(hydroxymethyl)aminomethane hydrochloride (Tris-HCl) (30 mM, 100 μL) at pH 8.0 in a screw-top vial. The solution was heated to 65 °C for 3 hours adding iodoalkane (5 μL) at 30 min intervals. The solvent was removed under reduced pressure, and the resulting dry solid was dissolved in ethylenediaminetetraacetic acid (EDTA) (0.1 M, 100 μL) at pH 8.0. The DNA was desalted using a NAP-25 column (GE Healthcare, NJ, USA). Fractions of 250 μL were collected, and the fractions containing DNA were identified by monitoring absorption at 260 nm. Alkyl-PPT ssDNA was purified using sodium dodecyl sulphate (SDS) polyacrylamide gel electrophoresis (PAGE) (10% pre-cast gel, Mini-Protean TGX, Bio-RAD). Alkyl-PPT modifications were characterized at ATD Bio by Ultra performance liquid chromatography - tandem mass spectrometry (UPLC-MS), using a Waters glycoprotein BEH Amide, 300 Å column.

1.3. Assembly of Hydrophobic DNA Duplexes

DNA duplexes were assembled by combining equimolar amounts of each ssDNA in PBS. The sample was heated at 65 °C for 5 min and cooled at 18 °C for 5 min using a BIORAD T100 Thermal Cycler. SDS-PAGE was used to validate dsDNA assembly. Running conditions: 140 V for 50 min and visualized by ethidium bromide staining. A 100 base pair ladder (NEB, UK) was used as a reference standard for migration. DNA sequences are shown in Table S1.

1.4. Size Determination Using Dynamic Light Scattering (DLS)

DLS experiments were performed on an Uncle instrument, Unchained Labs (CA,USA). Measurements were conducted at 20 °C on 0.5 mL samples in quartz cuvettes. For each sample, at least three measurements were performed. The acquisition time was 10s for each measurement, and at least 10 scans were run per measurement.

1.5. Analysis of DNA-Membrane Interaction in Vesicles using Fluorescence Microscopy

Preparation of GFP-encapsulated giant unilamellar vesicles (GUVs) were prepared using a published protocol.[3-4] GUVs were added to a MatTek dish and visualized using a Leica TCS CPE High-resolution Spectral Confocal Microscope, 63x/1.25 oil immersion objective. GUVs (5 μL) were incubated with Cy3-labeled DNA duplex (1 μM) in PBS for 5 minutes, and images were taken using laser excitations of 488 nm (GFP) and 532 nm (Cy3). Other buffer solutions were tested and include: Opti-MEM, and PBS supplemented with $MgCl_2$ (10 mM). All images were taken using the same exposure and gain settings. Cy3 membrane fluorescence was measured using ImageJ software, and statistical analysis was carried out using Prism (GraphPad, La Jolla, CA, USA). The intensity of the encapsulated GFP can

vary and have been adjusted for display purposes in figure 3. These levels were not adjusted before image analysis.

1.6. Atomistic Molecular Dynamics (MD) Simulations

All MD simulations were performed using program NAMD2,[5] a 2 fs integration time step, 2-2-6 multiple time stepping, periodic boundary conditions, and particle mesh Ewald (PME) method over a 1-Å resolution grid to calculate the long-range electrostatic interaction.[6] The Nose-Hoover Langevin piston[7] and Langevin thermostat were used to maintain the constant pressure and temperature in the system. An 8-10-12 Å cutoff scheme was used to calculate van der Waals and short-range electrostatic forces. SETTLE algorithm[8] was applied to keep water molecules rigid whereas RATTLE algorithm[9] constrained all other covalent bonds involving hydrogen atoms. CHARMM36 force field parameters described the bonded and non-bonded interactions among the atoms of DNA[10], lipid[11], water and ions. Custom corrections to non-bonded interactions were applied to improve description of ion-DNA[12], ion-ion, and DNA-lipid interactions.[13] The force-field parameters for the charge-neutral nucleotide variants were obtained using the CGenFF server.[14] Because of the lack of reliable parameters for alkyl-phosphorothioate, the modified nucleotides were built to contain alkyl-phosphate, not alkyl-phosphorothioate, chemical groups. Similar parameterization was used previously to characterize the structure, dynamics and ion conductance of ethyl-modified six-helix DNA bundle embedded in a lipid bilayer membrane.[15] We expect this parameterization choice to have minimal effect on the properties of the simulated systems and be negligible for the comparison of the effects of different alkyl group modifications. The coordinates of the system were saved every 20 ps. The visualization, analysis, and post-processing the simulation trajectories were performed using VMD[16] and CPPTRAJ.[17]

The atomic model of 30 base pair double-stranded (ds) DNA was built using the NAB module[18] of AMBERTOOLs according to the experimental nucleotide sequence. Starting from this initial model, six models of alkyl-modified dsDNA were created by replacing six consecutive base pairs, either at the center or at the terminal of the molecule, with their charge-neutral modifications containing either ethyl, butyl or hexyl phosphate groups at the DNA backbone. Following that, we inserted each alkyl-modified dsDNA normal to a 9 x 9 nm^2 patch of pre-equilibrated 1-palmitoyl-2-oleoyl-sn-glycero-3-phosphocholine (POPC) lipid bilayer membrane such that the alkyl modified region of the DNA was inside the hydrophobic region of the bilayer. To build a control system, we inserted an unmodified DNA into the lipid membrane in the same manner as the terminally modified DNA constructs. After inserting the DNA into the lipid membranes, the lipid molecules located within 2 Å of the DNA were removed. Twenty four Mg^{2+}-hexahydrates were placed near the DNA to neutralize its electrical charge. The resulting systems were solvated with TIP3P water molecules[19] using the solvate plugin of VMD. Sodium and chloride ions were added to a 150 mM concentration using the autoionize plugin of VMD. The final assembled systems measured approximately 9 x 9 x 14 nm^3 and contained approximately 87,000 atoms, see supplementary Fig. S1.

The assembled systems were first subject to energy minimization using the conjugate gradient method to remove steric clashes between the solute and the solvent. Following that, each system was equilibrated for 20 ns while harmonically restraining the phosphorus atoms of the DNA with a spring constant, k_{spring}, of 1 kcal mol^{-1} Å^{-2}, allowing the lipid molecules to equilibrate around the DNA. Subsequently, the harmonic restraints were released and the systems were equilibrated for 80 ns while reenforcing the hydrogen bonds within the base pairs using additional harmonic potentials (k_{spring} = 1 kcal mol^{-1} Å^{-2}). The systems were then equilibrated without any restraints for 1 μs each system in a constant number of atoms (N), pressure (P = 1 bar) and temperature (T = 298 K) ensemble. We used anisotropic pressure coupling to maintain the constant ratio of the system's dimension within the

membrane, allowing the system's dimension normal to the membrane to adjust independently of the other dimensions.

Steered molecular dynamics (SMD) method[20-21] was used pull terminally anchored DNA systems, T^{Et}, T^{Bt}, T^{Hex}, T^{Unmod} out of the membrane along the bilayer normal. In order to prevent the interactions across the periodic images of the molecules along the pulling direction, we reduced the size of the DNA construct to 15 base pairs by removing the DNA base pairs located away from the membrane. Each system was then equilibrated for 20 ns while harmonically restraining the phosphorus atoms of the DNA (k_{spring} = 1 kcal mol^{-1} Å^{-2}). Next, the systems were equilibrated for 30 ns while harmonically restraining the phosphorus atoms of the DNA and the C2 atoms of the lipids to their initial coordinates along the bilayer normal (k_{spring} = 0.01 kcal mol^{-1} Å^{-2}). The last frame of each 50 ns equilibration was used to initiate a 100 ns SMD simulations where the SMD force was applied to the center of mass of the terminal three base pairs embedded in the lipid membrane. The SMD template was moved with a constant velocity of 0.5 Å/ns and coupled to the DNA target with k_{spring} = 5 kcal/mol^{-1} Å^{-2}. These SMD simulations were performed while maintaining the harmonic restrains on the C2 atoms of lipids. Additionally, a tclforces script harmonically (k_{spring} = 1 kcal mol^{-1} Å^{-2}) restrained the phosphorous atom of the DNA to the surface of a cylinder (10 Å in radius), allowing the DNA to move coaxially along the direction of the SMD force.

1.7. Plasmid DNA Construction and Production of Stable MyrPalm-EGFP HeLa Cell Line

The retroviral packaging plasmid pCMS28-MyrPALM-EGFP-IRES-Puro was made by ligation of a gene synthesized DNA fragment (Thermo Fisher Scientific, GeneArt), containing a sequence encoding the amino acids MGCIKSKGKDS (MyrPalm, myristoylation and palmitoylation sequence of Lyn kinase) followed by an in-frame sequence of EGFP (Enhanced Green Fluorescent Protein). The MyrPalm sequence was flanked by 5' BglII and 3' EcoRI cloning sites. This fragment was ligated into pCMS28-EGFP-IRES-Puro[22] cut at BglII and EcoRI sites to make pCM28-MyrPalm-EGFP-IRES-Puro. The HeLa MyrPalm-EGFP expressing cell line was made by viral transduction using murine leukemia virus-based retroviruses as described previously.[22] Retroviral packing vector pCMS28-MyrPalm-EGFP-IRES-Puro was co-transfected with pVSVG (Addgene 12259) into HEK293-GP cells (ClonTech). Supernatants were collected, clarified by centrifugation (200 × g for 5 min), filtered (0.45 µm), and used to infect target HeLa cells at a multiplicity of infection (MOI) <1. The cell line was selected with Puromycin (Thermo Fisher Scientific) at final concentrations of 2.0 µg/mL. Clonal selected cell lines were grown on maintenance concentration of 0.5µg/mL Puromycin.

1.8. Cell Culture

MyrPalm-EGFP HeLa cells and wild type HeLa cells (a gift from Dr Jeremy Carlton, King's College London) were cultured in Dulbecco's Modified Eagle Medium (DMEM) (high glucose, GlutaMAX, pyruvate; Gibco; Life Technologies), supplemented with 10 % Fetal Bovine Serum (FBS) and 1 % Penicillin – Streptomycin in T-75 flasks. The culture was maintained in a humidified atmosphere containing 5 % CO_2 at 37 °C and passaged regularly every ~3 days.

1.9. Analysis of DNA-Membrane Interaction in Live Cells using Fluorescence Microscopy

MyrPalm-EGFP HeLa cells were plated at a density of 15,000 cells per well, in a chamber slide (Ibdi μ-Slide 8 Well) and incubated at 37 °C. The following day, the cells were washed with Opti-MEM and incubated with Cy3-labelled DNA duplex (0.2 µM , 300 µL) for 5 minutes. Cells were then washed three times with Opti-MEM to remove any non-internalized DNA. Images were acquired on Nikon Eclipse Ti inverted confocal microscope equipped with a Yokogawa CSU-X1 spinning disk, 20x air or 60x oil immersion objective at the Nikon Imaging Centre, KCL. The imaging system was equipped

with an environmental chamber to maintain cells at 37 °C and 5 % CO_2. Images were taken using laser excitations of 488 nm (GFP) and 532 nm (Cy3) and colocalized to determine any DNA-membrane interaction. All images were taken using the same exposure and gain settings. Image analysis was carried out using ImageJ and statistical analysis conducted using Prism. This protocol was also repeated in human bone osteosarcoma (U2OS) cells.

1.10. Nuclease Digestion of Membrane-Bound DNA Duplexes

The fluorescence Cy3 emission peaks of the DNA duplexes were monitored using a Varian Eclipse fluorescence spectrophotometer. The samples were analyzed by excitation at 540 nm, and following incubation with DNase I (20 U/mL) (NEB) with DNase I reaction buffer (NEB) for 10 minutes at 37 °C. The emission was scanned between 550 to 750 nm. A 5–10 mm slit width and 600–800 PMT voltage were used, and quartz cuvettes with a path length of 1 cm. The maximum intensity of the Cy3 peak after treatment with DNase was normalized to the wavelength before addition of DNase, to determine the change in Cy3 fluorescence.

DNA digestion by DNase I was analyzed by gel electrophoresis. DNA duplex (0.2 μM) was treated with DNase I (20 U/mL) (NEB) in DNase I reaction buffer (NEB) for 10 minutes at 37 °C. The digestion was terminated by placing the sample on ice and adding loading dye containing 1% SDS and 10 mM EDTA to inhibit DNase activity. Treated and untreated DNA samples were loaded onto an agarose gel (2%) and run in Tris-acetate-EDTA (TAE) buffer at 60 V for 50 min. A 100 base pair ladder was used as a reference standard for migration. The gel was visualized using a fuji FLA series fluorescence gel imager equipped with a 532 nm excitation source. The Cy3 bands were analyzed in ImageJ to determine the intensity and thus % of Cy3 co-localized with DNA fragments. The gel was later visualized by ethidium bromide staining.

MyrPalm-EGFP HeLa cells were incubated with Cy3-labelled DNA duplex as above. Following incubation and washing with Opti-MEM, the cells were incubated with DNase I (20 U/mL) (NEB) with DNase I reaction buffer (NEB) for 10 minutes. The cells were washed and re-suspended in Opti-MEM and imaged. All images were taken using the same exposure and gain settings. Image analysis was carried out using ImageJ software and statistical analysis conducted on Prism.

1.11. Sphingomyelinase treatment of DNA-bound cells.

MyrPalm-EGFP HeLa cells were incubated with DNA duplexes as above. Following incubation and washing with Opti-MEM, the cells were incubated with Sphingomyelinase (SMase) from Bacillus cereus (Merck) in PBS at 0.5 U for 30 min. The cells were washed and re-suspended in Opti-MEM and imaged. All images were taken using the same exposure and gain settings. Image analysis was carried out using ImageJ software and statistical analysis conducted on Prism.

2. Supplementary Tables and Figures

Table S1. DNA sequences of hydrophobic DNA duplexes

Name	DNA	Sequence 5′ → 3′
T^{Chol}	ss_1	TTG CTA CGT G TA CCG TAT CGT TAG ACC GAT /Cy3
	ss_2	ATC GGT CTA ACG ATA CGG TAC ACG TAG CAA /CholTEG
C PPT	ss_1	TTG CTA CGT GTA C*C*G* T*A*T* CGT TAG ACC GAT /Cy3
	ss_2	ATC GGT CTA ACG A*T*A* C*G*G* TAC ACG TAG CAA
T PPT	ss_1	T*T*G* C*T*A* CGT GTA CCG TAT CGT TAG ACC GAT /Cy3
	ss_2	ATC GGT CTA ACG ATA CGG TAC ACG* T*A*G* C*A*A
Nat	ss_1	TTG CTA CGT G TA CCG TAT CGT TAG ACC GAT /Cy3
	ss_2	ATC GGT CTA ACG ATA CGG TAC ACG TAG CAA

Table S2. Size determination and polydispersity % of hydrophobic DNA duplexes using dynamic light scattering.

DNA Duplex	Mean size (nm)	Polydispersity (%)
Nat	5.5 ± 1.5	32
T^{Chol}	7.2 ± 2	28
T^{Et}	5.7 ± 2	35
C^{Et}	5.5 ± 1.9	35
T^{Hex}	5.9 ± 2	35
C^{Hex}	5.8 ± 1.6	28

Table S3. Details of simulated systems:

Equilibrium simulations of 30-bp dsDNA in POPC membrane			SMD simulations of 15-bp dsDNA pulling out of POPC membrane		
System	*Number of atoms*	*Simulation time*	*System*	*Number of atoms*	*Simulation time*
C^{Et}	83376	1.00 μs	T^{Et}	86973	0.11 μs
C^{Bt}	82701	1.02 μs	T^{Bt}	87105	0.11 μs
C^{Hex}	83865	1.05 μs	T^{Hex}	87039	0.10 μs
T^{Et}	85117	1.03 μs	$T^{unmodified}$	86868	0.10 μs
T^{Bt}	84418	1.00 μs	Equilibrium simulations of 15-bp dsDNA in aqueous solution		
T^{Hex}	86251	1.13 μs			
$T^{unmodified}$	85399	0.41 μs	T^{Et}	48137	0.09 μs
			T^{Bt}	48068	0.30 μs
			T^{Hex}	48119	0.11 μs
			$T^{unmodified}$	37755	0.10 μs

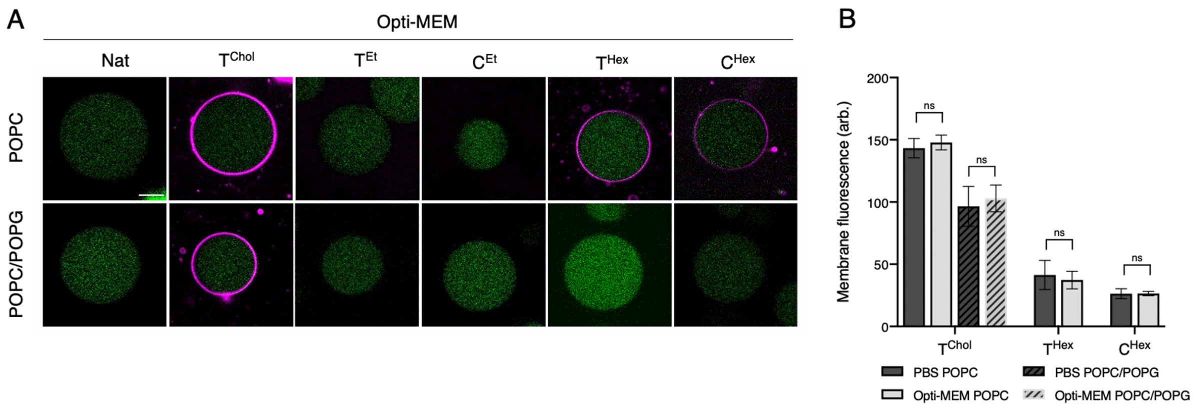

Figure S1. DNA-membrane interaction with POPC and 1:1 POPC/POPG GUVs in Opti-MEM cell culture medium or PBS (A) Fluorescence confocal microscopy analysis of dsDNA (1 μM) in Opti-MEM buffer with GFP-encapsulated GUVs following 5 min incubation. GUVs are composed of POPC or 1:1 POPC/POPG lipids. The panels present the overlay of GFP (green) and Cy3 (purple) channels. Representative images from 3 independent experiments are shown. The intensity of the encapsulated GFP can vary and has been adjusted for display purposes. The Cy3 images were collected and processed identically. Scale bar, 10 μm. (B) A plot summarizing the relative Cy3-DNA membrane fluorescence intensities in PBS (dark gray) and Opti-MEM (light gray) buffers on the membranes of POPC and 1:1 POPC/POPG GUVs (striped pattern). Analysis of DNA in Opti-MEM as in panels A, and DNA in PBS as in figure 3. The data is presented as mean ± SD collected from three independent experiments, n = 10 GUVs per condition. Two-way ANOVA using Sidak's multiple comparisons test, ns= not significant.

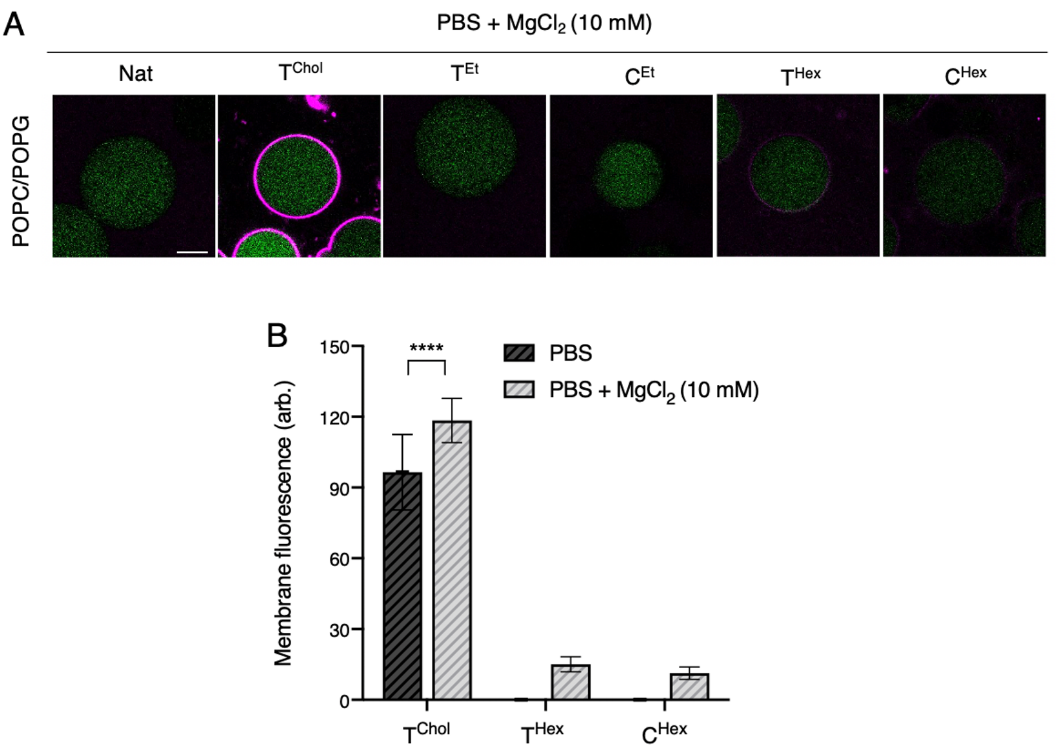

Figure S2. DNA-membrane interaction in negatively charged synthetic membranes is increased in the presence of $MgCl_2$ ions. (A) Fluorescence confocal microscopy analysis of dsDNA (1 μM) in PBS buffer supplemented with $MgCl_2$ (10 mM) and GFP-encapsulated GUVs following 5 min incubation. GUVs are composed of 1:1 POPC/POPG lipids. The panels present the overlay of GFP (green) and Cy3 (purple) channels. Representative images from 3 independent experiments are shown. The intensity of the encapsulated GFP can vary and has been adjusted for display purposes. The Cy3 images were collected and processed identically. Scale bar, 10 μm. (B) A plot summarizing the relative Cy3-DNA membrane fluorescence intensities of DNA in PBS (figure 3) or PBS supplemented with $MgCl_2$ (10 mM) as in panels A. The data is presented as mean ± SD collected from three independent experiments, n = 10 GUVs per condition. Two-way ANOVA using Sidak's multiple comparisons test (****$P < 0.0001$).

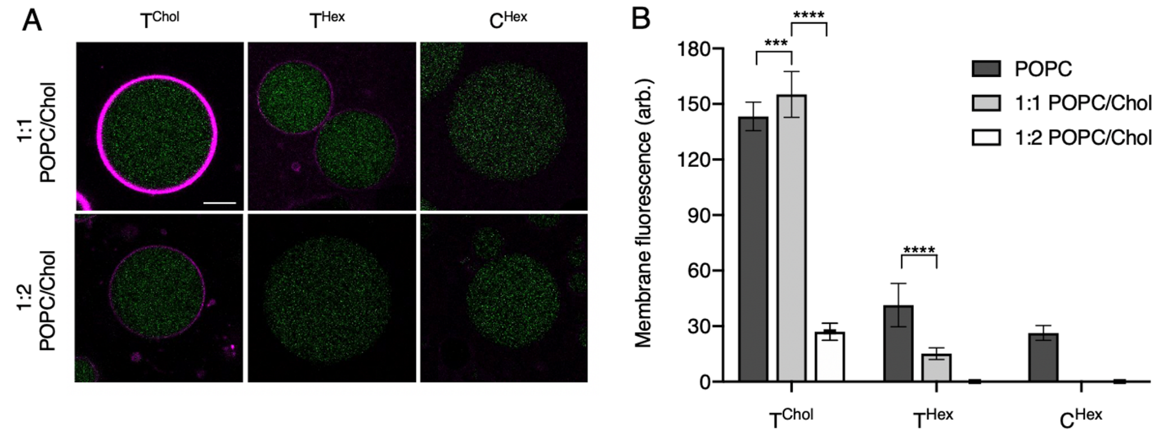

Figure S3. DNA-membrane interaction is influenced by the cholesterol content in POPC synthetic membranes. (A) Fluorescence confocal microscopy analysis of dsDNA (1 µM) in PBS buffer and GFP-encapsulated GUVs, composed of either 1:1 POPC/Cholesterol or 1:2 POPC/Cholesterol lipids, following 5 min incubation. The panels present the overlay of GFP (green) and Cy3 (purple) channels. Representative images from 3 independent experiments are shown. The intensity of the encapsulated GFP can vary and has been adjusted for display purposes. The Cy3 images were collected and processed identically. Scale bar, 10 µm. (B) A plot summarizing the relative Cy3-DNA membrane fluorescence intensities of DNA in PBS in POPC (dark gray) as in figure 3, and 1:1 POPC/Chol (light gray), 1:2 POPC Chol (white) as in panels A. The data is presented as mean ± SD collected from three independent experiments, n = 10 GUVs per condition. Two-way ANOVA using Tukey's multiple comparisons test (***P = 0.0004, ****P < 0.0001).

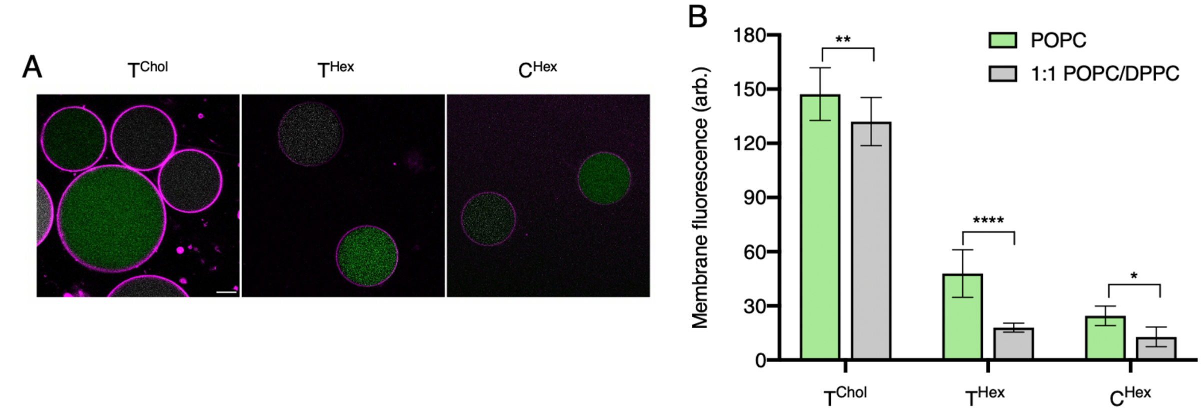

Figure S4. DNA-membrane interaction in POPC and 1:1 POPC/DPPC synthetic membranes (A) Fluorescence confocal microscopy analysis of dsDNA (1 µM) in PBS buffer and GFP-encapsulated GUVs, composed of either POPC (green) or 1:1 POPC/DPPC lipids, following 5 min incubation. The panels present the overlay of GFP (green) and Cy3 (purple) channels. Representative images from 3 independent experiments are shown. The intensity of the encapsulated GFP can vary and has been adjusted for display purposes. The Cy3 images were collected and processed identically. Scale bar, 10 µm. (B) A plot summarizing the relative Cy3-DNA membrane fluorescence intensities of DNA in PBS in POPC (green) and 1:1 POPC/DPPC (gray). The data is presented as mean ± SD collected from three independent experiments, n = 10 GUVs per condition. Two-way ANOVA using Sidak's multiple comparisons test (*P = 0.0397, **P = 0.0047, ****P < 0.0001).

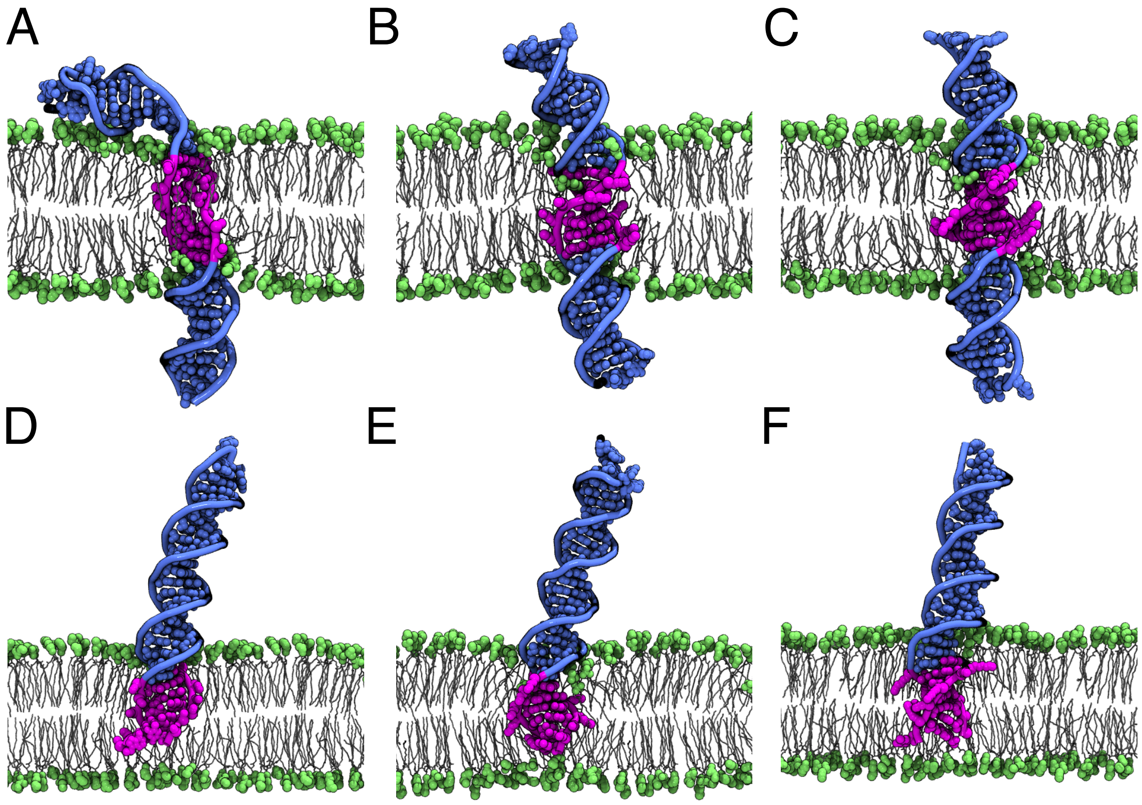

Figure S5 Configurations of the simulation systems after 1μs equilibrium MD run. Cut-away view of the microscopic configurations of the centrally anchored ethyl (A), butyl (B) and hexyl (C) phosphate modified DNA at the end of the 1 μs MD run. Cut-away view of the microscopic configurations of the terminally anchored ethyl (D), butyl (E) and hexyl (F) phosphate modified DNA at the end of the 1 μs MD run. The unmodified DNA bases are shown in blue whereas the modified nucleotides are shown in magenta. The DNA backbone is shown as thin tubes and the base atoms are represented as spheres. Non-hydrogen atoms of the amine-phosphate lipid headgroups are shown as green spheres whereas the lipid tails are shown as white lines, water and ions not shown for clarify.

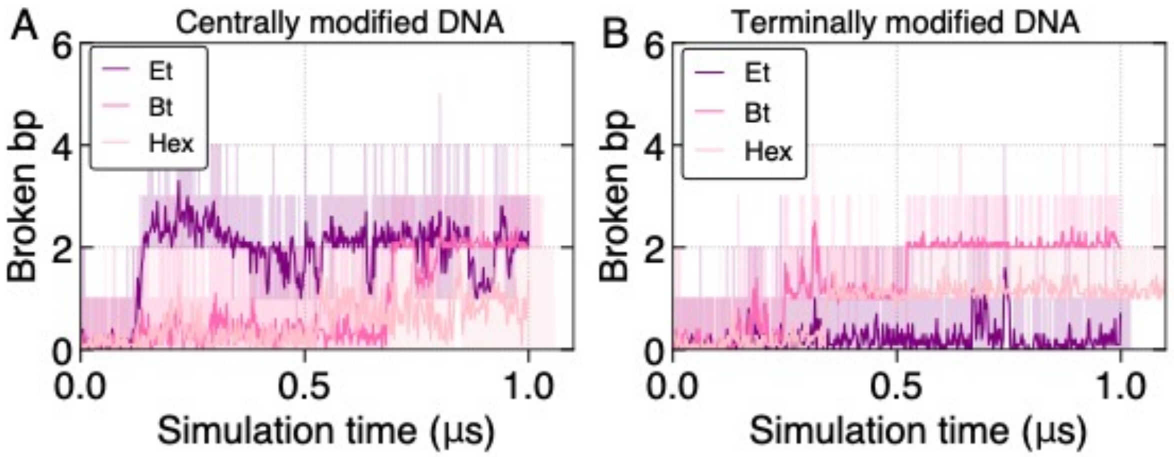

Figure S6. The number of the broken base-pairs in the modified DNA fragment as a function of simulation time for centrally (A) and terminally (B) anchored systems.

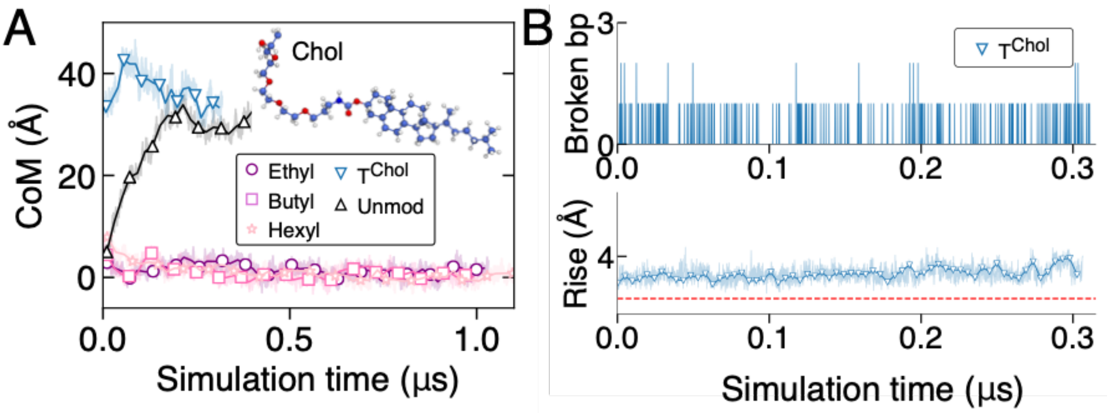

Figure S7. MD simulation of dsDNA fragments terminally anchored to the membrane. (A) CoM *z*-coordinate of the six terminal DNA base pairs tethered to a lipid bilayer as a function of simulation time. The inset illustrates the chemical attachment of the cholesterol anchor to a DNA terminal. The unmodified DNA escapes from the membrane within the first 200 ns of the simulation. The ethyl, butyl and hexyl system data are the same as presented in Figure 4b. The cholesterol data are reproduced from Arnott et al.[23] Note that in the MD simulation of the cholesterol-conjugated DNA, the DNA remained tethered to the lipid bilayer membrane through a chemically linked cholesterol moiety that was embedded within the membrane. (B) The number of broken base pairs (top) and the average rise (bottom) of the terminal six base pairs of the cholesterol-tethered dsDNA construct during the free equilibration simulation.

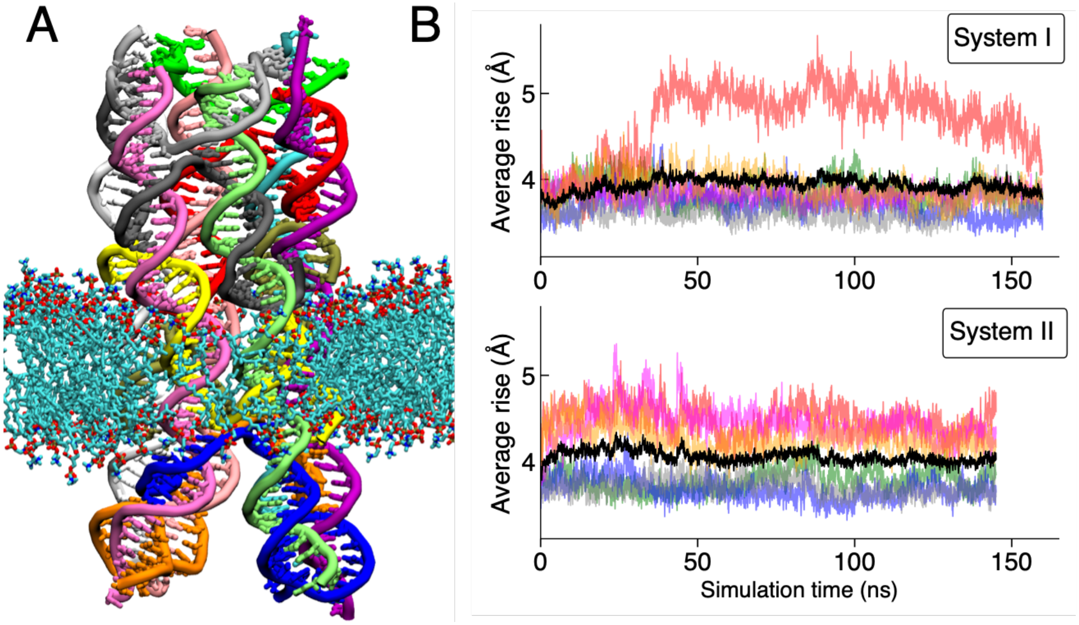

Figure S8. Local stretching of ethyl-modified DNA in a membrane-embedded six helix DNA bundle. (A) An illustration of a six helix DNA nanopore embedded in a DPhPC lipid bilayer membrane taken from a previous molecular dynamics study.[15] Two types of the DNA design were investigated in that study: one where all of the membrane spanning DNA nucleotides carried charged-neutral modifications at the backbone (system I) and another where DNA modifications were introduced only to those DNA nucleotides that were facing the lipid membrane (system II). (B) Average rise of the ethyl-modified fragment of each DNA duplex of the channel (color). The black line shows the rise of the modified fragments averaged over the six helices of the DNA nanopore.

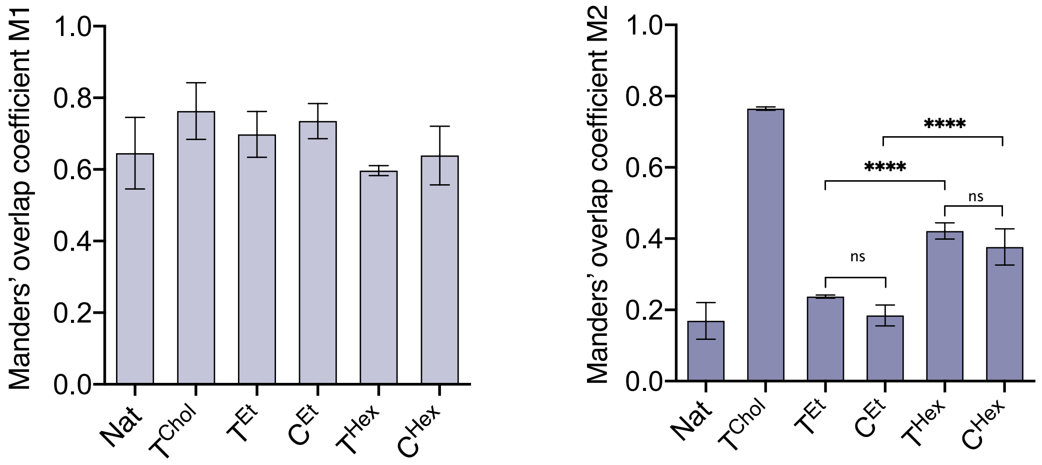

Figure S9. Manders' coefficient to determine co-localization and homogeneity of DNA distribution at the plasma membrane. M1 is the fraction of overlap of Cy3 on GFP and M2 in the fraction of overlap of GFP on Cy3. M2 can be used to determine the distribution and homogeneity of Cy3 relative to the GFP plasma membrane. One-way ANOVA, Tukey's multiple comparisons test between each condition (****$P < 0.0001$).

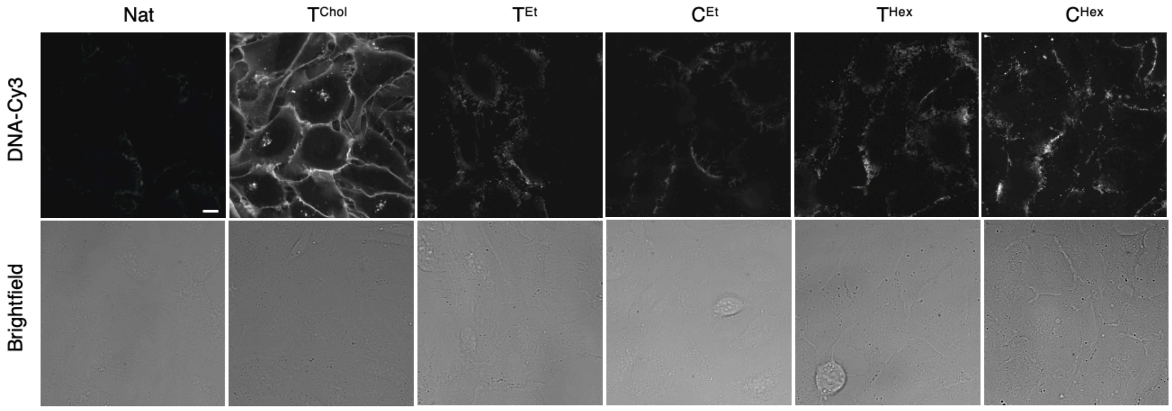

Figure S10. Hydrophobic DNA duplexes bind to the plasma membrane of live U2OS cells and demonstrate anchor-specific interactions. Confocal fluorescence microscopy images of live U2OS cells treated with hydrophobic dsDNA (0.2 μM) in Opti-MEM for 5 min. The fluorescently labeled DNA (top panels) and brightfield (bottom panels) are shown. Representative images from 3 independent experiments are shown, n = 300 cells analyzed per condition. All images were collected and processed under identical conditions. Scale bar, 10 μm.

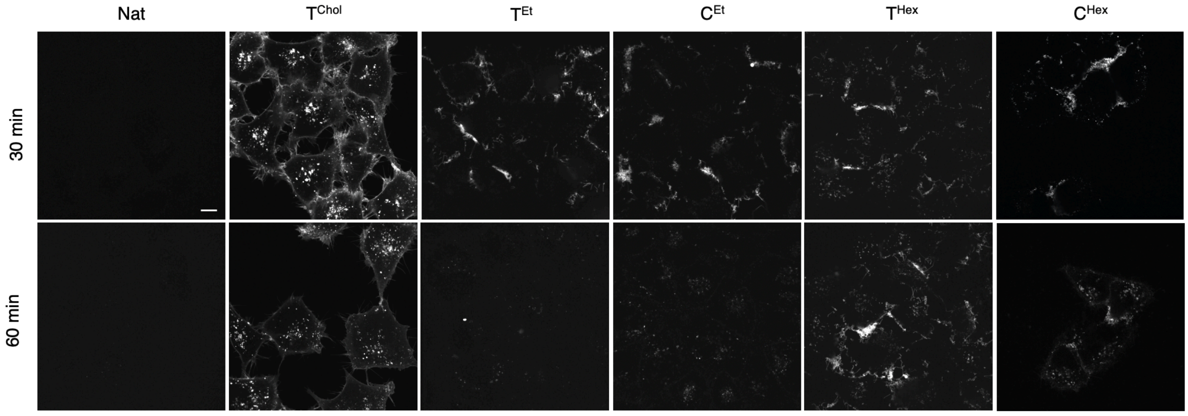

Figure S11. DNA dwell time at the plasma membrane and DNA internalization into live HeLa is influenced by hydrophobic anchor type. Confocal fluorescence microscopy images of live HeLa cells treated with hydrophobic dsDNA (0.2 μM) in Opti-MEM for 5 min, washed, and imaged after 30 min and 60 min. Representative images from 3 independent experiments are shown, n = 300 cells analyzed per condition. All images were collected and processed under identical conditions. Scale bar, 10 μm.

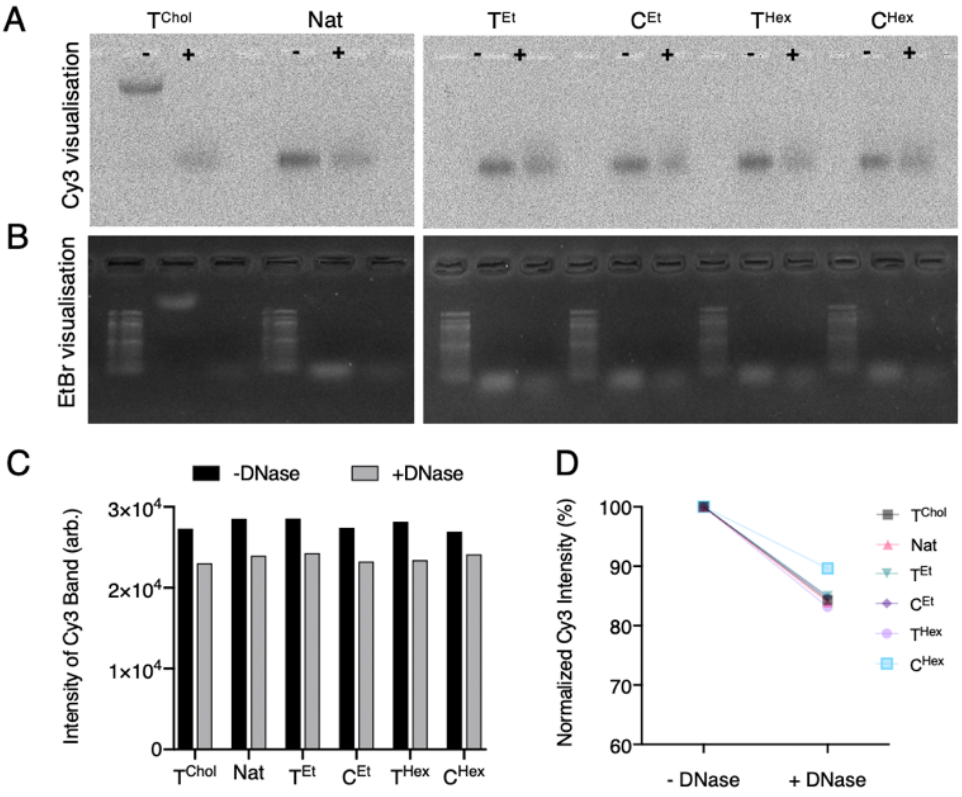

Figure S12. Treatment of hydrophobic DNA duplexes with DNase I. (A) and (B) show an agarose gel (2%) of the DNA duplexes untreated (-) and treated (+) with DNase I (20 U/mL) (NEB) with DNase I reaction buffer (NEB) for 10 minutes at 37 °C. Following incubation with DNase, the vials were placed on ice and DNA loading dye and SDS solution was added (contains 1% SDS and 10 mM EDTA to inhibit DNase activity). The untreated samples (-) were also incubated and ran in the same solution, but without addition of DNase I. The starting concentration of DNA is 0.2 μM, in accordance with the DNA concentration used in the cell-based nuclease digestion experiment in figure 8. Panel A show the agarose gel imaged using a fuji FLA series fluorescence gel imager equipped with a 532 nm excitation source. The gel was later stained by EtBr and re-imaged as seen in (B). 100 base pair ladder is used a standard reference for migration. (C) Bar chart show the intensity of the Cy3 bands from (A) measured using ImageJ. (D) Plot shows the normalized Cy3 intensity (%) from (C) to demonstrate change in Cy3 intensity as a result of DNase digestion, and the fraction of Cy3 that co-localize with fragmented DNA.

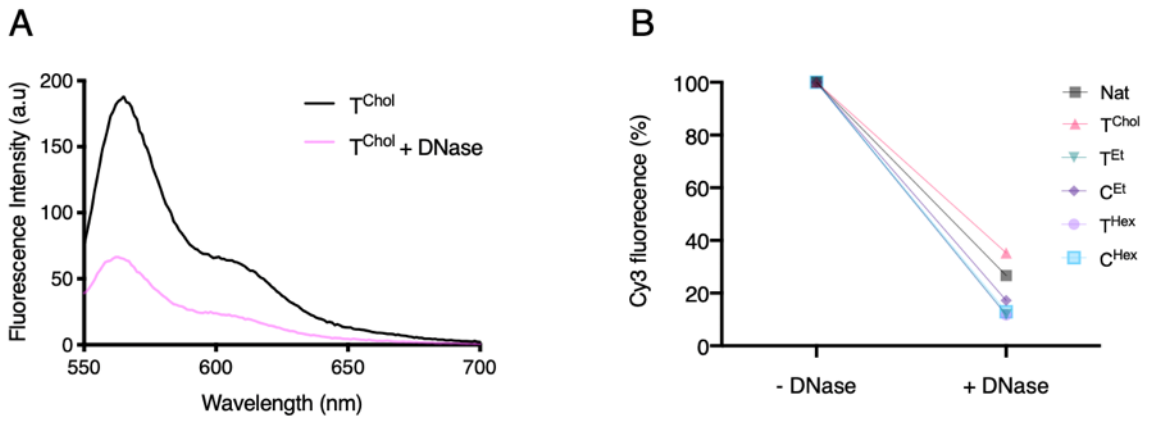

Figure S13. Treatment of hydrophobic DNA duplexes with DNase I. (A) Fluorescence emission spectra of Cy3-labeled T^{Chol} before (black) and after (magenta) addition of DNase I. T^{Chol} shown as an example. (B) Plot showing the normalized change in Cy3 emission fluorescence intensity following addition of DNase I for all DNA duplexes.

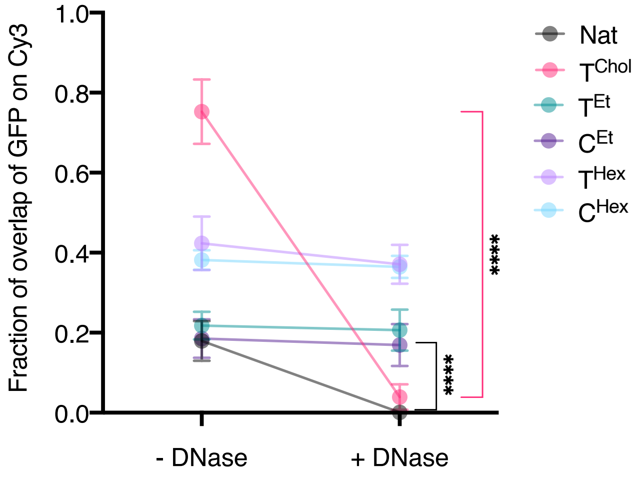

Figure S14. Manders' coefficient to determine change in the fraction of overlap of GFP following DNase addition. Analysis of the nuclease digestion confocal microscopy data in figure 5. Two-way ANOVA, Sidak's multiple comparisons test comparing Manders' coefficient per condition, with and without DNase addition (****P < 0.0001)

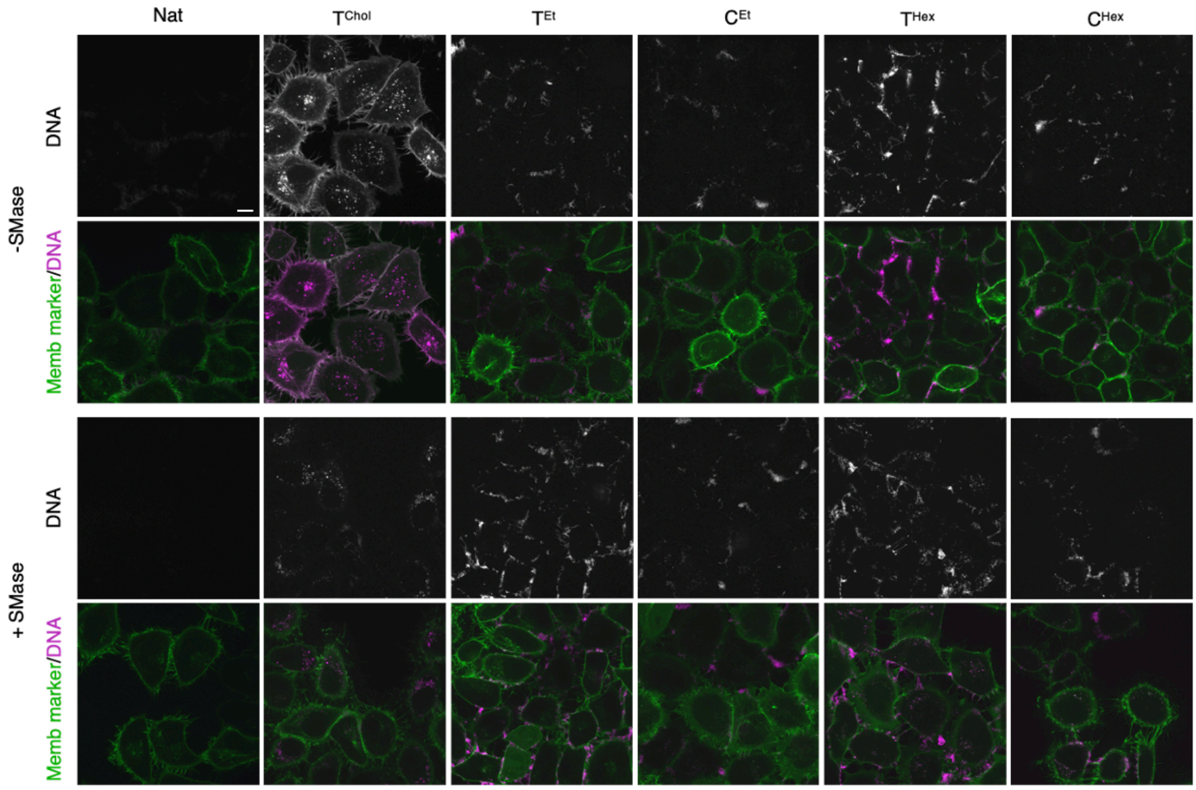

Figure S15. Sphingomyelinase digestion in the exoplasmic leaflet of the membrane influences membrane-tethered duplexes but has no effect on membrane-spanning duplexes. MyrPalm-EGFP HeLa cells were incubated with DNA for 5 min and washed with Opti-MEM. Confocal fluorescence microscopy images were taken of control cells kept in PBS for 30 min (-SMase) (A) and cells treated with SMase (0.5 U) in PBS for 30 min (+SMase) (B). The top panels, in both A and B, show the DNA and the bottom panels show the overlay of DNA and membrane marker (DNA is magenta and the membrane marker is green). Representative images from 3 independent experiments are shown. All images were collected and processed under identical conditions. Scale bar, 10 μm.

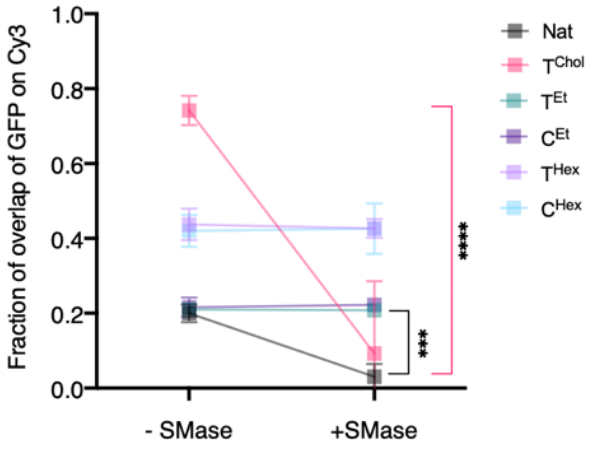

Figure S16. Manders' coefficient determines the fraction of overlap of GFP on membrane-bound DNA for the sphingomyelinase digestion assay. Analysis of confocal microscopy data in figure X, control cells that are untreated (-SMase) and cells treated with SMase (+SMase) for 30 min. Two-way ANOVA, Sidak's multiple comparisons test comparing Manders' coefficient per condition, with and without SMase addition (****P < 0.0001, ***P = 0.0008).

3. Captions to Supplementary Movies

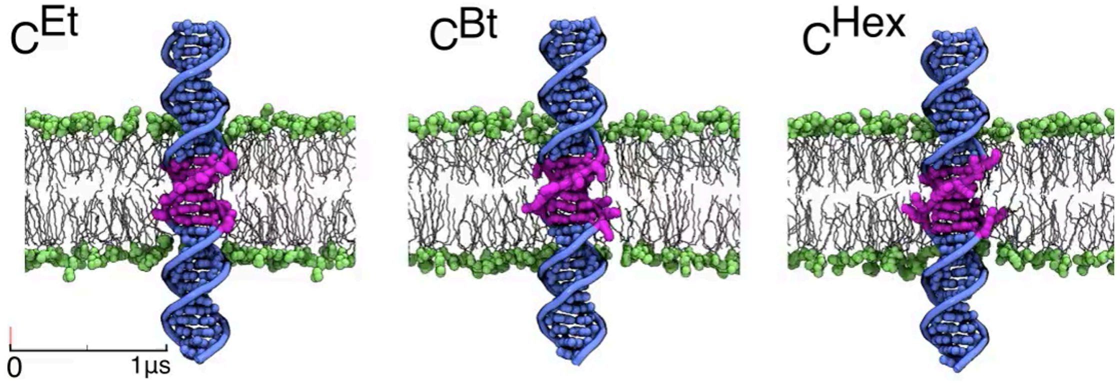

Supplementary Movie 1. All-atom molecular dynamics simulation of ethyl, butyl and hexyl modified dsDNA molecules centrally anchored to a POPC lipid bilayer membrane. The movie shows a cut-away view of 1 μs long equilibration trajectories for three separate systems. The unmodified and unmodified DNA nucleotides are shown in blue and magenta respectively. The DNA backbone is shown as thin tubes whereas nucleotide base atoms are represented using vdW spheres. Non-hydrogen atoms of the amine-phosphate lipid headgroups are shown as green spheres whereas the lipid tails are shown as white lines. Water and ions are not shown for clarity.

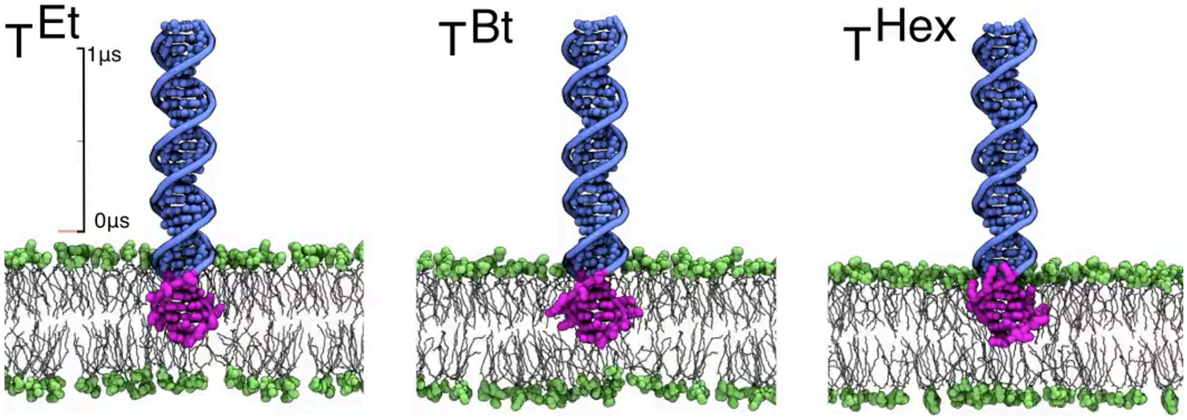

Supplementary Movie 2. All-atom molecular dynamics simulation of terminally modified ethyl, butyl and hexyl dsDNA molecules anchored into the POPC lipid bilayer membrane. The movie shows a cut-away view of 1 μs long equilibration trajectories for three separate systems. The unmodified and unmodified DNA nucleotides are shown in blue and magenta respectively. The DNA backbone is shown as thin tubes whereas nucleotide base atoms are represented using vdW spheres. Non-hydrogen atoms of the amine-phosphate lipid headgroups are shown as green spheres whereas the lipid tails are shown as white lines. Water and ions are not shown for clarity.

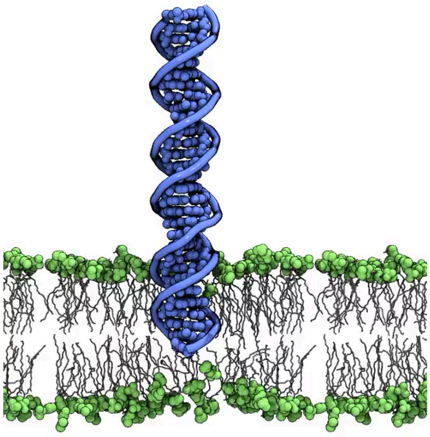

Supplementary Movies 3. All-atom MD simulation of unmodified DNA initially anchored in a POPC lipid bilayer membrane. The DNA is seen to escape from the membrane in less than 400 ns, the simulation duration. The DNA backbone is shown as thin blue tubes whereas nucleotide base atoms are represented using vdW spheres. Non-hydrogen atoms of the amine-phosphate lipid headgroups are shown as green spheres whereas the lipid tails are shown as white lines. Water and ions are not shown for clarity.

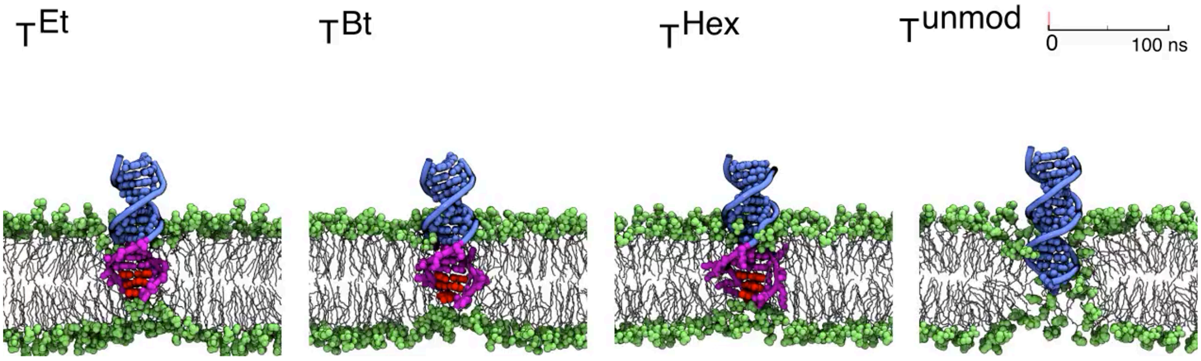

Supplementary Movies 4. SMD pulling of terminally anchored DNA constructs from a lipid bilayer membrane. The movie illustrates cut-away view of four separate SMD simulations each being 100 ns long. The unmodified and unmodified DNA nucleotides are shown in blue and yellow, respectively. The DNA backbone is shown as thin tubes whereas nucleotide base atoms are represented using vdW spheres. Non-hydrogen atoms of the amine-phosphate lipid headgroups are shown as green spheres whereas the lipid tails are shown as white lines. Water and ions are not shown for clarity.

4. References

1. Burns, J. R.; Stulz, E.; Howorka, S., Self-assembled DNA nanopores that span lipid bilayers. *Nano Lett.* **2013,** *13*, 2351-2356.

2. Burns, J. R.; Al-Juffali, N.; Janes, S. M.; Howorka, S., Membrane-spanning DNA nanopores with cytotoxic effect. *Angew. Chem.* **2014,** *53*, 12466-12470.

3. Burns, J. R.; Howorka, S., Defined bilayer interactions of DNA nanopores revealed with a nuclease-based nanoprobe strategy. *ACS Nano* **2018,** *12*, 3263-3271.

4. Krishnan, S.; Ziegler, D.; Arnaut, V.; Martin, T. G.; Kapsner, K.; Henneberg, K.; Bausch, A. R.; Dietz, H.; Simmel, F. C., Molecular transport through large-diameter DNA nanopores. *Nat. Commun.* **2016,** *7*, 1-7.

5. Phillips, J. C.; Hardy, D. J.; Maia, J. D.; Stone, J. E.; Ribeiro, J. V.; Bernardi, R. C.; Buch, R.; Fiorin, G.; Hénin, J.; Jiang, W., Scalable molecular dynamics on CPU and GPU architectures with NAMD. *J. Chem. Phys.* **2020,** *153*, 1-33.

6. Batcho, P. F.; Case, D. A.; Schlick, T., Optimized particle-mesh ewald/multiple-time step integration for molecular dynamics simulations. *J. Chem. Phys.* **2001,** *115*, 4003-4018.

7. Feller, S. E.; Zhang, Y.; Pastor, R. W.; Brooks, B. R., Constant pressure molecular dynamics simulation: the langevin piston method. *J. Chem. Phys.* **1995,** *103*, 4613-4621.

8. Miyamoto, S.; Kollman, P. A., Settle: An analytical version of the SHAKE and RATTLE algorithm for rigid water models. *J. Comput. Chem.* **1992,** *13*, 952-962.

9. Andersen, H. C., Rattle: A "velocity" version of the shake algorithm for molecular dynamics calculations. *J. Comput. Phys.* **1983,** *52*, 24-34.

10. Hart, K.; Foloppe, N.; Baker, C. M.; Denning, E. J.; Nilsson, L.; Mackerell Jr, A. D., Optimization of the CHARMM additive force field for DNA: improved treatment of the BI/BII conformational equilibrium. *J. Chem. Theory Comput.* **2011,** *8*, 348-362.

11. Klauda, J. B.; Venable, R. M.; Freites, J. A.; O'connor, J. W.; Tobias, D. J.; Mondragon-Ramirez, C.; Vorobyov, I.; Mackerell Jr, A. D.; Pastor, R. W., Update of the CHARMM all-atom additive force field for lipids: validation on six lipid types. *J. Phys. Chem. B* **2010,** *114*, 7830-7843.

12. Yoo, J.; Aksimentiev, A., Improved parametrization of Li+, Na+, K+, and Mg2+ ions for all-atom molecular dynamics simulations of nucleic acid systems. *J. Phys. Chem. Lett.* **2012,** *3*, 45-50.

13. Yoo, J.; Aksimentiev, A., Improved parameterization of amine–carboxylate and amine–phosphate interactions for molecular dynamics simulations using the CHARMM and AMBER force fields. *J. Chem. Theory Comput.* **2015,** *12*, 430-443.

14. Vanommeslaeghe, K.; Hatcher, E.; Acharya, C.; Kundu, S.; Zhong, S.; Shim, J.; Darian, E.; Guvench, O.; Lopes, P.; Vorobyov, I., CHARMM general force field: a force

field for drug-like molecules compatible with the CHARMM all-atom additive biological force fields. *J. Comput. Chem.* **2010,** *31*, 671-690.

15. Yoo, J.; Aksimentiev, A., Molecular dynamics of membrane-spanning DNA channels: conductance mechanism, electro-osmotic transport, and mechanical gating. *J. Phys. Chem. Lett.* **2015,** *6*, 4680-4687.

16. Humphrey, W.; Dalke, A.; Schulten, K., VMD: Visual molecular dynamics. *J. Mol. Graph. Model.* **1996,** *14*, 33-38.

17. Case, D. A.; V. Babin; J.T. Berryman; R.M. Betz; Q. Cai; D.S. Cerutti; T.E. Cheatham, I.; T.A. Darden; R.E. Duke; H. Gohlke; A.W. Goetz; S. Gusarov; N. Homeyer; P. Janowski; J. Kaus; I. Kolossváry; A. Kovalenko; T.S. Lee; S. Legrand; T. Luchko; R. Luo; B. Madej; K.M. Merz; F. Paesani; D.R. Roe; A. Roitberg; C. Sagui; R. Salomon-Ferrer; G. Seabra; C.L. Simmerling; W. Smith; J. Swails; R.C. Walker; J. Wang; R.M. Wolf; X.Wu; Kollman, P. A., *AMBER 14*. University of California, San Francisco., 2014.

18. Macke, T. J.; Case, D. A. In *Modeling unusual nucleic acid structures*, ACS Symposium Series, Leontis, N. B.; Santalucia Jr, J., Eds. ACS Publications: 1998; pp 379-393.

19. Jorgensen, W. L.; Chandrasekhar, J.; Madura, J. D.; Impey, R. W.; Klein, M. L., Comparison of simple potential function for simulating liquid water. *J. Chem. Phys.* **1983,** *79*, 926-935.

20. Grubmüller, H.; Heymann, B.; Tavan, P., Ligand binding: molecular mechanics calculation of the streptavidin-biotin rupture force. *Science* **1996,** *271*, 997-999.

21. Isralewitz, B.; Izrailev, S.; Schulten, K., Binding pathway of retinal to bacterio-opsin: a prediction by molecular dynamics simulations. *Biophys. J.* **1997,** *73*, 2972-2979.

22. Carlton, J. G.; Martin-Serrano, J., Parallels between cytokinesis and retroviral budding: a role for the ESCRT machinery. *Science* **2007,** *316*, 1908-1912.

23. Arnott, P. M.; Joshi, H.; Aksimentiev, A.; Howorka, S., Dynamic interactions between lipid-tethered DNA and phospholipid membranes. *Langmuir* **2018,** *34*, 15084-15092.
